# Noise Reduction by Diffusional Dissipation in a Minimal Quorum Sensing Motif

**DOI:** 10.1371/journal.pcbi.1000167

**Published:** 2008-08-29

**Authors:** Yu Tanouchi, Dennis Tu, Jungsang Kim, Lingchong You

**Affiliations:** 1 Department of Biomedical Engineering, Duke University, Durham, North Carolina, United States of America; 2 Department of Electrical and Computer Engineering, Duke University, Durham, North Carolina, United States of America; 3 Institute for Genome Sciences and Policy, Duke University, Durham, North Carolina, United States of America; California Institute of Technology, United States of America

## Abstract

Cellular interactions are subject to random fluctuations (noise) in quantities of interacting molecules. Noise presents a major challenge for the robust function of natural and engineered cellular networks. Past studies have analyzed how noise is regulated at the intracellular level. Cell–cell communication, however, may provide a complementary strategy to achieve robust gene expression by enabling the coupling of a cell with its environment and other cells. To gain insight into this issue, we have examined noise regulation by quorum sensing (QS), a mechanism by which many bacteria communicate through production and sensing of small diffusible signals. Using a stochastic model, we analyze a minimal QS motif in Gram-negative bacteria. Our analysis shows that diffusion of the QS signal, together with fast turnover of its transcriptional regulator, attenuates low-frequency components of extrinsic noise. We term this unique mechanism “diffusional dissipation” to emphasize the importance of fast signal turnover (or dissipation) by diffusion. We further show that this noise attenuation is a property of a more generic regulatory motif, of which QS is an implementation. Our results suggest that, in a QS system, an unstable transcriptional regulator may be favored for regulating expression of costly proteins that generate public goods.

## Author Summary

Quorum sensing (QS) is a mechanism by which many bacteria regulate gene expression via the synthesis and detection of small, diffusible signals. Since its discovery, QS has been shown to control diverse physiological functions in numerous types of bacteria. It provides an elegant strategy for bacteria to sense their density and to achieve coordinated population behavior. By stochastic modeling, we show that QS can effectively reduce variability (“noise”) in the expression of its target genes. Surprisingly, the noise reduction does not significantly depend on the number of bacteria but rather results from the coupling of a bacterium and its environment through signal diffusion. Diffusion enables fast signal turnover, which, together with fast intracellular turnover of the cognate receptor of the signal, leads to noise reduction. Our work suggests a unique role of QS in achieving robust gene regulation, which is distinct from noise-regulation mechanisms that act at the intracellular level. As such, it offers novel insights into evolution of QS as well as its application in construction of synthetic gene circuits.

## Introduction

Cellular processes are subject to random fluctuations (or noise) in quantities of interacting molecules. Cells may take advantage of noise to achieve diverse functions [1],[2]. In a mechanism called stochastic resonance, noise may improve detection of weak periodic input signals [3], whereas stochastic focusing may turn a gradual response into a threshold-like response [4]. Also, noise is often exploited to initiate cell differentiation or phenotypic switching. A classical example is the lysis-lysogeny decision in phage λ development, where noise is necessary to trigger the decision [5]. Similarly, noise is implicated in the competence development of *Bacillus subtilis* [6].

However, noise often presents a major challenge for reliable cellular function. To this end, cells use specific biochemical networks or motifs to minimize deleterious effects of noise [1],[7]. For instance, reducing translation burst rates (number of proteins synthesized per transcript) attenuates noise in gene expression [8]. Based on this observation, it has been argued that evolution tends to favor noise reduction in essential genes as they appear to have smaller burst rates compared with non-essential genes in yeast [9]. Also, several regulatory motifs have been found to be effective in reducing noise. Negative feedback reduces noise by shifting the noise spectrum to a higher frequency region [10]–[12]. Ultrasensitive switches and feedforward loops are able to attenuate noise in input signals [13],[14].

These noise regulation mechanisms all operate at the intracellular level. At the population level, cell–cell communication may play an important role in achieving robust gene expression dynamics. For example, it has been shown to be important for the proper function of many rhythmic processes in physiology [15],[16]. Intuitively, intracellular noise, which primarily originates from the stochastic nature of chemical reactions of interacting species and fluctuations in cellular conditions, may be reduced when a population of cells carries out their function cooperatively.

**Figure 1:**
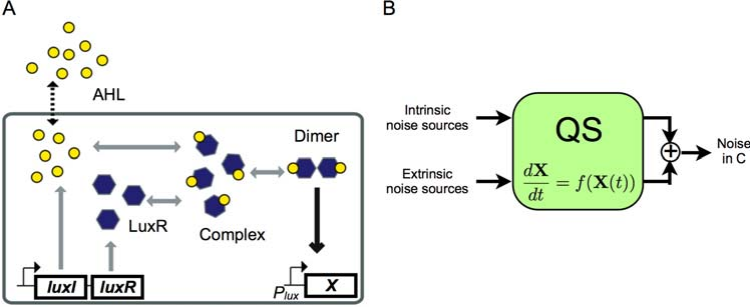
QS system. (A) A minimal QS motif. AHL and LuxR are produced at constant rates inside the cell. AHL can diffuse across the cell membrane. At sufficiently high concentrations, the intracellular AHL binds to and activates LuxR. The active LuxR (the complex) further dimerizes and activates a downstream gene (*X*) controlled by the *P_lux_* promoter. Many natural QS systems share this common motif. (B) Noise processing by QS. Noise sources can be considered as inputs to the system. A QS motif processes the noise sources and results in noise in *C* as an output.

In bacteria, cell–cell communication can be established by quorum sensing (QS), a mechanism by which many bacteria broadcast and sense their density [17]–[19]. A canonical QS system is the *lux* system from the marine bacterium *Vibrio fischeri* (Figure 1A). This system consists of two genes encoding proteins LuxI and LuxR. LuxI is an AHL (acyl homoserine lactone) synthase; LuxR is a transcriptional regulator activated by the AHL. The AHL signal is produced inside the cell but freely diffuses across the cell membrane into the environment: therefore, the AHL concentration is low at a low cell density. As the cell density increases, the signal accumulates in the environment and inside the cell. At sufficiently high concentrations, AHL can bind to and activate LuxR, which will then activate downstream genes. Lux-type QS systems are common in gram negative bacteria [18],[20], and they are critical for regulating diverse physiological functions, such as bioluminescence, biofilm formation, and bacterial pathogenicity [18],[20],[21].

In this study, we use mathematical modeling to analyze noise regulation in QS-mediated gene expression. Recently, stochastic simulations of QS-mediated dynamics have been carried out for *Vibrio fischeri* [22] and *Agrobacterium tumefaciens* [23]. These studies have provided insights into dynamics of QS-regulated gene expression coupled with positive feedback regulation and population dynamics. In contrast, however, we have focused on a minimal QS motif without feedback regulation, in order to dissect the contribution of QS per se to noise regulation. Also, it should be noted that not all QS systems have feedback regulation. For instance, Ravn et al identified that production of 3-Oxo-hexanoyl-homoserin lactone (OHHL, one type of AHL signal) in *Serratia proteamaculans* (SprI/R system) and *Erwinia carotovora* (CarI/R or ExpI/R system; both CarI and ExpI produce OHHL) is approximately constitutive [24].

We find that QS can serve as an effective noise-reduction mechanism. In particular, diffusion of the QS signal and fast decay of the transcriptional regulator can reduce noise by synergistically attenuating low-frequency components of extrinsic noise. We term this noise reduction mechanism “diffusional dissipation,” as its defining feature is fast dissipation of signal molecules through diffusion. Further analysis indicates that this noise attenuation is the result of a more generic regulatory motif—bimolecular interaction—of which QS is an implementation. Our results further suggest a connection between QS-regulated functions and the decay of the QS transcriptional regulator. An unstable transcriptional regulator may be favored for regulating expression of costly proteins that generate public goods, as QS-mediated noise reduction in the target protein can increase the average population fitness, thus providing a selection advantage.

## Results

### Model Development

To analyze noise regulation by QS, we develop a simple kinetic model (Materials and Methods and Text S1) to account for the reactions illustrated in Figure 1A. In order to isolate and examine the role of the communication feature of the QS system per se, we omit additional feedback regulation of signal synthesis. Briefly, we assume constitutive production of the QS signal (*A*) and the LuxR protein (*R*), which interact to form a complex (*C*). The complex further forms homodimers to activate a target gene (*X*) controlled by the *lux* promoter. We omit the dimerization process and focused on the fluctuations of the complex at steady state, although incorporation of the dimerization does not seem to change our conclusion (Figure S1). Also, to simplify analysis and to gain deeper insight, we focus on a single cell that is coupled with its extracellular environment by signal production, diffusion, and detection. Numerical simulations indicate that noise regulation by multiple cells (coupled by QS) is similar to that by a single cell (Figure S2).

In this model, we consider two types of noise sources: (1) intrinsic noise source, which arises from the stochastic nature of chemical reactions in the QS system (Table S1); and (2) extrinsic noise source, which originates from fluctuations in cellular machinery outside the QS system (Materials and Methods). While its exact origin remains unclear, extrinsic noise is a major component of the total noise in bacterial systems [25]–[27]. Intuitively, we can consider QS system as a signal processing module that takes noise sources as inputs and transmits them to *C* as noise (Figure 1B).

### Diffusion Reduces Extrinsic Noise

We first examine the effect of diffusion on noise in *C* by varying the diffusion rate constant (*P*) from 0 (no diffusion) to 2×10^−11^ L min^−1^. The diffusion rate constants of glucose and lactose through the outer membrane of wild type *E. coli* have been experimentally estimated to be 3.6×10^−12^ and 1.8×10^−13^ L min^−1^, respectively [28]. Also, the diffusion rate constants of sugars show a strong dependence on their molecular weights [28]. Although AHL is not a member of the sugar group, we first apply the same dependence on AHL to obtain an estimate of its diffusion rate constant. Accordingly, 3-oxo-hexanoyl-homoserine lactone (3OC6HSL), the AHL signal produced by *Vibrio fischeri*, is estimated to be ∼2×10^−12^ L min^−1^. Different QS modules may use different AHLs that have different diffusion rate constants. Thus we set the fastest diffusion rate constant as 2×10^−11^ L min^−1^, which is 10-fold larger than the estimated value. We then modulate the production rate constant of *A* to “balance” changes in the diffusion rate constant, in order to maintain the same steady-state level of *C*. By doing so, we aim to reveal noise modulation in *C* due to different diffusion rate constants that is otherwise masked by changes in average protein levels.

**Figure 2:**
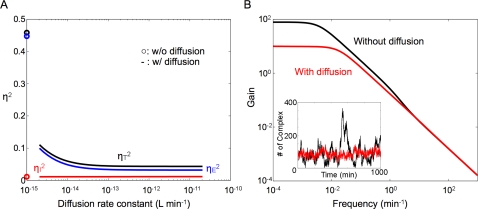
Diffusion reduces the output noise by reducing the extrinsic noise component. (A) Diffusion drastically reduces the total noise (black) by primarily attenuating the extrinsic noise (blue) without significantly affecting the intrinsic noise (red). Circles represent levels of total (black), extrinsic (blue) and intrinsic (red) noise without diffusion. (B) Diffusion significantly reduces the gain of the low-frequency extrinsic noise components transmitted to the complex. *P* = 2×10^−13^ L min^−1^ for the with-diffusion case. Inset: The corresponding time courses of the complex without diffusion (black) and with diffusion (red). Numerical simulation is implemented using the fixed time step 4^th^ order Runge-Kutta method [56] for the deterministic terms and Euler-Maruyama method for the stochastic terms.

Figure 2A demonstrates that the presence of diffusion drastically reduces total noise in *C* 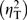. This reduction increases with an increasing diffusion rate constant: 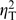 is reduced by 90% as the diffusion rate constant increases from 0 to 2×10^−13^ L min^−1^ (numeric simulation shows ∼80% reduction. See Materials and Methods and Text S1 for more discussion). The reduction of total noise is evident in time courses of *C* for the two cases (Figure 2B, inset). Decomposition of the total noise reveals that this noise reduction is primarily due to reduction of the extrinsic noise (Figure 2A). The intrinsic noise in *C* 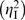 actually increases slightly (<0.2%) for the same changes in the diffusion rate constant. Close inspection of 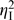 indicates that some intrinsic noise sources increase but the others decrease (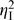 is a sum of contributions of intrinsic noise sources from different reactions) with increasing diffusion rate constants (Figure S3).

Frequency analysis provides further insights into the noise reduction mechanism. The transfer function of the extrinsic noise source (|*H^ξ^*(*f*)|, Materials and Methods) shows that the extrinsic noise becomes band-limited by the QS system. With diffusion (*P* = 2×10^−13^ L min^−1^), the gain of low-frequency components (*f*<0.02 min^−1^) decreases by about 8-fold (Figure 2B). There is a slight but negligible increase in the gain of high-frequency components (*f*>7.9 min^−1^). In essence, diffusion effectively reduces extrinsic noise by reducing the transmission of fluctuating signals (including noise) in the low-frequency domain.

### Fast *R* Decay Reduces Extrinsic Noise

In a *lux*-type QS system, the R protein is often highly unstable in the absence of its cognate signal [29]–[31]. It is seemingly a waste of energy for bacteria because a faster *R* decay rate constant would require faster production to maintain the same *R* level, everything else being equal. Here we investigate whether the noise may be affected by different *R* decay rate constants (*γ_R_*). Again, the steady-state level of *C* was maintained by modulating the production of *R*.

**Figure 3:**
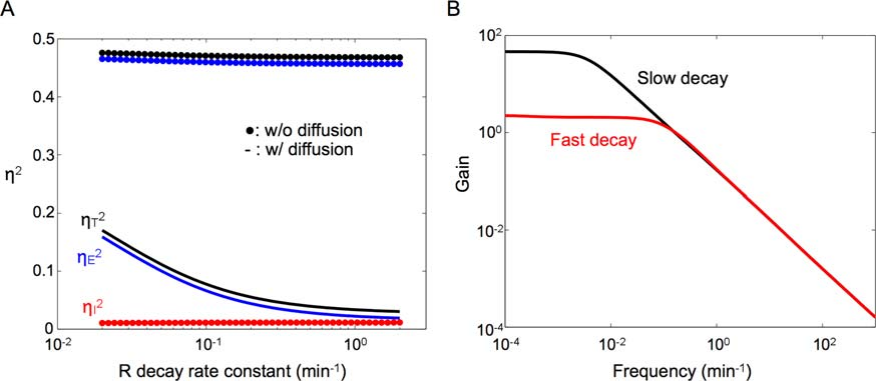
Fast *R* decay reduces the extrinsic noise in the presence of fast signal diffusion. (A) With diffusion (*P* = 2×10^−13^ L min^−1^), faster *R* decay drastically reduces total noise (black line) by decreasing the extrinsic noise (blue line) without significantly affecting the intrinsic noise (red line). Without diffusion, faster *R* decay has little effect on either the intrinsic or the extrinsic noise (dotted lines). (B) Fast *R* decay significantly reduces the gain of low-frequency extrinsic noise components transmitted to the complex in the presence of diffusion. For these calculations, *P* = 2×10^−13^ L min^−1^, *γ_R_* = 0.02 min^−1^ for slow *R* decay, and 2 min^−1^ for fast *R* decay.

We find that faster *R* decay results in much more reduction in the extrinsic noise of *C* than in its intrinsic noise in the presence of diffusion (Figure 3A). For *P* = 2×10^−13^ L min^−1^, 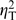 decreases by more than 80% when *γ_R_* varies from 0.02 to 2 min^−1^ (solid black line). However, the noise reduction becomes negligible (<2%) in the absence of diffusion (dotted black line). This result indicates a synergistic coupling between signal diffusion and faster decay of *R* in reducing cellular noise. That is, the noise reduction by increased *γ_R_* is enhanced by larger *P* and vice versa. In contrast, intrinsic noise is not significantly affected (<1%) with or without diffusion (red solid or dotted line).

Again, we examine how the transfer function of the extrinsic noise is affected by increasing *γ_R_*. |*H^ξ^*(*f*)| shows the similar tendency to the case of diffusion (Figure 3B). In the presence of diffusion, fast *R* decay (*γ_R_* = 2 min^−1^) reduces the gain of low-frequency components by ∼20-fold compared with the case with slow *R* decay (*γ_R_* = 0.02 min^−1^). Similar to fast diffusion (Figure 2B), fast *R* decay causes slight but negligible increase in the gain of high-frequency noise components (*f*>0.16 min^−1^, Figure 3B).

### QS as an Implementation of a More Generic Regulatory Motif

**Figure 4:**
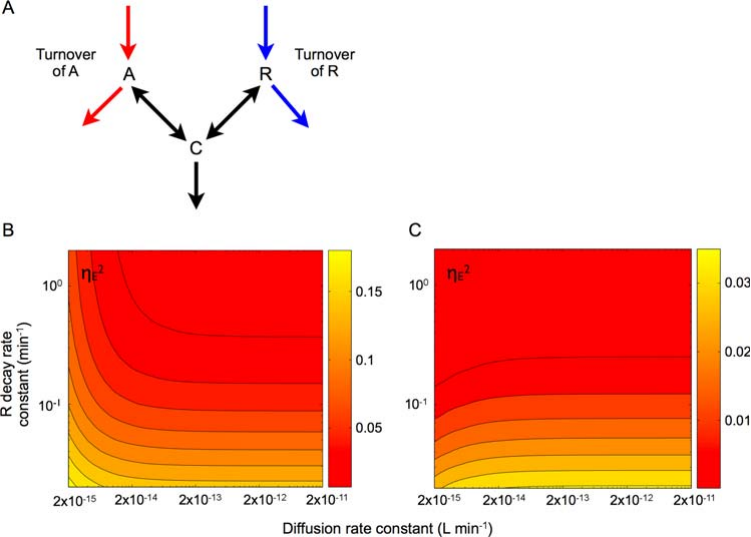
The QS module represents an implementation of a more general regulatory motif. (A) This motif entails constant production and decay of *A* and *R*, which bind reversibly to form *C*, the system output. Fast signal diffusion in the QS system corresponds to a fast turnover in *A*. (B) Assuming identical extrinsic noise inputs into *A*, *R* and *C*, faster turnover in *A* and *R* synergistically reduces the extrinsic noise in *C*. (C) If only *R* is directly affected by the extrinsic noise, faster turnover in *A* results in a slight increase in the extrinsic noise in *C*.

As shown in Figures 2B and 3B, the behavior of the low frequency component plays an important role in extrinsic noise attenuation. This characteristic can be captured by a simpler system (Figure 4A) when 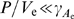 by treating the export of *A_i_* by diffusion as effective decay (Text S1). This simplification makes intuitive sense as fast signal diffusion and fast decay of *R* would have the same qualitative consequence: they both increase the turnover of the corresponding cellular component. We set a new decay rate constant of *A*, 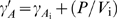 while maintaining the steady state of each molecule the same as before the simplification by modulating the production rate constant of *A* (other parameters remain the same). As expected, fast turnover of *A* and *R*, which corresponds to fast dissipation of *A* and *R*, synergistically reduces extrinsic noise in *C* when the condition is satisfied (Figure 4B, *P*<2×10^−14^ L min^−1^). Also, we show analytically that the DC component (or the low-frequency components whose behavior can be approximated by the DC component) of the transfer function of extrinsic noise sources decreases monotonically as the turnover of *A* and *R* becomes faster (Text S1). Interestingly, even when the condition is not satisfied (*P*>2×10^−14^ L min^−1^), 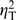 differs by less than 3% from the original model (data not shown). Therefore, in this framework, the QS system can be considered as a special case of a structurally symmetric regulatory motif.

### Parameter Dependence

Overall, our results are insensitive to variations in the base parameters, except for those that characterize extrinsic noise sources (Text S1 and Figure S4). We have so far assumed that different extrinsic noise sources were fully correlated and identical for all species (*A_i_* (or *A* in the simplified model), *R* and *C*). However, this assumption may not always hold in a real system. In an extreme case, when the extrinsic noise completely arises from *R*, increasing turnover of *A* results in an increase in the extrinsic noise in *C* (Figure 4C). In fact, faster turnover of *A* increases extrinsic noise originating from *R* while that of *R* reduces it. Thus, if turnover of both molecules becomes fast enough, we still see a significant noise reduction in *C* (Figure 4C, from lower left corner to upper right corner). The opposite is also true: faster turnover of *R* increases extrinsic noise from *A* while that of *A* reduces it (data not shown). As actual magnitudes of extrinsic noise sources to each species are unclear, we cannot exclude the possibility of unbalanced extrinsic noise sources exemplified above. However, the framework of our analysis is still able to account for these alternative scenarios.

In this study, we have assumed the extrinsic noise sources to be white. This assumption may appear at odds with experimental observations, which suggest that extrinsic noise is band-limited by cell division [25]. However, according to our results, QS-mediated reduction of the extrinsic noise happens for low-frequency components. As such, assuming band-limited extrinsic noise sources will not change our conclusions. We have also assumed perfect correlation between the extrinsic noise sources. For arbitrary correlation, we examine 10,000 randomly generated combinations of correlations and find that ∼95% of them exhibit the synergistic noise reduction in *C* by fast diffusion and *R* decay (Text S1 and Figure S5).

## Discussion

Extensive studies have been carried out to define characteristics of noise generation [8], [26], [32]–[36], propagation [13],[25],[27],[37], and regulation (negative feedback [10]–[12], ultrasensitivity [13], and feedforward loop [14]). Complementary to those mechanisms that operate at the intracellular level, quorum sensing may serve as an additional layer of control for regulating robust cell behavior. On one hand, it may facilitate synchronization of complex dynamics generated by otherwise independent circuits in a population of cells [38]–[40], or enable generation of coherent population dynamics by integration of cell populations [41]–[44]. On the other, it may directly modulate the noise characteristics in individual cells. To this end, Cox et al analyzed stochastic dynamics of QS in *V. fischeri* in two aspects: the role of positive feedback and the modulation of noise frequencies by reversible reactions [22]. By analyzing a minimal QS motif without feedback regulation, our study aims to expose the contribution to noise reduction by communication per se. To simplify analysis, we focus on noise reduction in the complex (*C*), which, upon dimerization, leads to activation of downstream genes. We find that fast diffusion and fast *R* decay can synergistically reduce the extrinsic noise in *C* but has relatively little impact on its intrinsic noise. The noise reduction is achieved by decreasing the gain of low-frequency fluctuations in the extrinsic noise.

Importantly, our analysis reveals that QS is a unique mechanism to attenuate extrinsic noise, which we call diffusional dissipation to underscore the importance of the signal and the R protein turnover. The term “diffusional” reflects fast signal turnover achieved by diffusion, a defining feature of AHL-based QS. Moreover, our analysis suggests that QS is an implementation of a more generic regulatory motif (Figure 4) in which the fast dissipation of two species (*A* and *R*), which together form a heterodimer (*C*), reduces extrinsic noise by suppressing low frequency components (Text S1); the signal diffusion in QS is a specific implementation to increase effective signal dissipation.

The importance of *A* and *R* turnover also explains the counterintuitive observation that noise reduction by multiple cells (coupled by QS) is similar to that by a single cell (Figure S2). Although the coupling of a cell with its environment via diffusion effectively speeds up *A_i_* turnover, further coupling with other cells does not significantly affect turnover of either *A_i_* or *R*. Also, although coupling among multiple cells reduces noise of extracellular AHL (*A_e_*) by increasing the total number of molecules, this reduction is unlikely to impact downstream gene expression, as the noise in *A*_e_ is already much smaller than that in other species even for a single cell (Figure S2).

How does the noise reduction in *C* affect downstream gene expression? In general, unbranched gene expression machinery (e.g., linear cascade) works as a low-pass filter whose critical frequency is largely determined by the decay rate constant of the output protein [11],[45]. Thus, high-frequency fluctuations in *C* will be filtered out by downstream genes. This makes modulation of low-frequency fluctuations in *C* particularly relevant. In fact, only reduction in low-frequency fluctuations can effectively affect downstream gene expression. Also, given that dimerization of *C* confers cooperativity to the downstream gene expression, fluctuation of *C* around the intermediate level could have a significant effect on gene expression [27]. As extrinsic noise often dominates total noise in gene expression [25],[26], noise reduction in *C* can thus effectively attenuate noise in downstream gene expression.

In a *lux*-type QS system, an R protein is often highly unstable in the absence of its cognate signal [29]–[31]. Fast R protein turnover may prevent premature activation of QS genes [46] or improve fidelity in recognizing the cognate signal (Smith, Song, and You, Signal discrimination by differential regulation of protein stability in quorum sensing, submitted). Our results suggest yet another scenario: this apparently wasteful process may facilitate reduction of extrinsic noise in the QS-regulated gene expression. However, under what conditions is reduction of extrinsic noise beneficial to cells? We note that QS often controls functions costly to individual but beneficial to the population. Examples include secretion of exoenzymes [47]–[49], production of antibiotics and exotoxins [18],[21], as well as bioluminescence [50]. In these scenarios, the benefit of the effector to an individual cell is determined by its total level in the population, whereas its cost to each cell solely depends on its expression rate in the cell. Therefore, noise in effector expression can lead to significant variations in the division rates of individual cells. If the cost of the effector increases with its level more than linearly, mathematical analysis indicates that reduction in the effector noise can increase the average fitness of the population (Figure S6). That is, in cooperative production of a common good, noise attenuation by QS might provide an intrinsic mechanism to resist decline of average population fitness and invasion of cheaters. In contrast, not all QS-regulated functions may benefit from noise reduction. However, as signal diffusion is essential for communication, noise reduction originating from fast turnover of the signal appears to be an indivisible side effect of QS systems. Again, it is worth emphasizing the noise modulation by the stability of R proteins. It would be interesting to explore a potential connection between the stability of R proteins and QS-regulated functions in the context of noise regulation.

## Materials and Methods

As detailed in Text S1, we model the system using the following coupled stochastic differential equations:
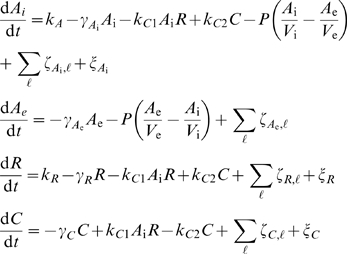
(1)where *A*_i_, *A*_e_, *R*, and *C* are numbers of the intracellular signal, the extracellular signal, the R protein, and the complex, respectively; 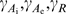, and *γ_C_* are their decay rate constants; *k_A_* and *k_R_* are production rate constants of *A_i_* and *R*; *P* is diffusion rate constant of the signal across the cell membrane; *V*_e_ is the average extracellular volume per cell; and *V*_i_ is an intracellular volume. Parameters for the base case are either obtained or estimated from literature (Table S2). 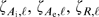 and *ζ_C_*_,*ℓ*_ denote intrinsic noise sources affecting respective species with the index *ℓ* specifying the reaction from which noise originates (Table S1). *ξ_A_*, *ξ_B_*, and *ξ_C_* denote extrinsic noise sources. Each intrinsic noise source is implemented as a multiplicative noise term whose magnitude depends on the instantaneous rate of the corresponding reaction. Each extrinsic noise source is additive and its magnitude is fixed (see Text S1 for details). Because we consider the extrinsic noise sources as fluctuations in intracellular machinery that influence the QS system, the equation for *A*_e_ does not contain a *ξ* term.

We assume that extrinsic noise sources are fully correlated with the same magnitude 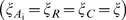 and their spectra are white. In reality, these extrinsic noise terms may be less correlated despite the fact that fluctuations in protein degradation machinery, intracellular pH or stochasticity in growth and cell division rates likely have global effects on all intracellular molecules. For instance, *A*_i_ is produced by an enzymatic reaction mediated by LuxI, so extrinsic noise sources for *A*_i_ may encompass fluctuations in LuxI or its substrate whereas those for *R* are possibly fluctuations in mRNA, RNA polymerase or ribosome (note that while extrinsic noise sources may be complex in reality, we use a lumped parameter, *ξ_m_*, to represent such possibly complex effect). Also, extrinsic noise sources may be rather band limited than white [25],[51]. We shall discuss the consequence of relaxing these simplifying assumptions in Discussion.

The system is monostable in the deterministic domain and does not exhibit noise-induced bistability (Text S1 and Figure S8) as observed in some other systems [52]. We are interested in the fluctuation of *C* around its steady state. To obtain the steady-state fluctuation, we first linearize the equations (Equation 1) and calculate the power spectral density (PSD) of noise in each species by solving them in the Fourier domain. The PSD of the noise in *C* (*S*(*f*)) is:
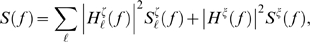
where *f* is frequency; 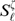 and *S^ξ^* are PSD of intrinsic and extrinsic noise sources contributing to noise in *C*; 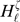 and *H^ξ^* are transfer functions of corresponding noise sources and they are inherent properties of the reaction network. Each noise source is individually processed by its corresponding transfer function, and transmitted to *C* (Figure 1B). In other words, each transfer function shows how the corresponding noise source is modulated in the frequency domain. According to Plancherel's theorem, integration of the PSD (*S*(*f*)) over the entire frequencies allows us to calculate variance of each species in the temporal domain [53]. The Langevin approach enables us to relate intrinsic and extrinsic noise in species of interest as following [27]:
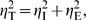
where 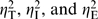 are total noise, intrinsic noise, and extrinsic noise, respectively.

Our analytical approach is based on the linearization of the system. The underlying assumption of linearization is that the noise is small enough so that the distribution of each molecule is sufficiently tight around the point of the linearization (corresponding to its steady state in a deterministic model). To test this assumption, we perform numerical simulations with the base parameter set and different diffusion rate constants and *R* decay rate constants (Figure S7 and Figure S8). We find that the linearization is valid for all cases except for *P* = 0 L min^−1^ (no diffusion). In this case, the distribution of *C* becomes wide and skewed, resulting in deviation from the small-noise regime and a discrepancy between its average and the corresponding deterministic steady state level (Figure S8). However, our analytical approach overall captures the qualitative trend of 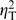 (including the synergistic effect of diffusion and *R* decay; not shown).

When only considering the intrinsic noise, we also note that the Langevin approach can recover the same PSD and related statistics as the chemical master equation approach for linear or linearized systems even when numbers of interacting molecules are small, as long as linearization can be justified [54]. Extrinsic noise sources cannot be implemented in master equations unless they are represented by additional explicit reactions. This is a part of the reason why we have employed the Langevin approach. However, when the number of molecules becomes too small, the assumptions required for accurate linearization may be violated (Text S1). Also, linearization might overlook possible resonance/band-pass filtering effects resulting from nonlinearity of the dimerization reaction [55]. However, our simulation results obtained from the nonlinear equations (Equation 1) indicate such nonlinear effects do not seem to be occurring in our system, at least for the base parameter set with varying *P* and *γ_R_* (0≤*P*≤2×10^−11^ L min^−1^, 0.02≤*γ_R_*≤0.2 min^−1^; not shown).

## Supporting Information

Text S1 (0.23 MB PDF)

Figure S1 Impact of the dimerization reaction. Noise and PSD in *D* are calculated using the analytical approach with the base parameter set (Table S2). (A) Extrinsic noise in *D* for varying *P* and *γ_R_*. Total noise in *D* shows the same dependence as the extrinsic noise is dominant (not shown). (B) The gain of extrinsic noise sources decreases as *γ_R_* increases (from the black line (*γ_R_* = 0.02 min^−1^) to the red line (*γ_R_* = 2 min^−1^)).
(0.06 MB PDF)

Figure S2 Simulation results of noise in *A*_i_, *A*_e_, *R*, and *C* under different coupling conditions: (1) each cell has its own microenvironment (e.g. no coupling). (2) 100 cells are divided into 10 populations each of which contains 10 cells coupled to one another via their environment, and (3) 100 cells form 1 population of 100 coupled cells. The conditions are indicated on the *x*-axis. The simulation is carried out as in Figure 2. Noise is calculated for individual cell from time course simulations for a span of 1,000 min (10,000 data points). Noise values shown are the average of 100 cells. For these calculations, *P* = 2×10^−13^ L min^−1^.
(0.02 MB PDF)

Figure S3 PSD of each intrinsic noise arising from the corresponding intrinsic noise source (*ζ*_1_, *ζ*_2_, …, *ζ*_9_). *P* is changed from 0 (black line) to 2×10^−13^ L min^−1^ (blue line). Accordingly, the PSDs of *ζ*_1_, *ζ*_3_, *ζ*_4_, *ζ*_7_, and *ζ*_8_ increase, while those of the other noise sources decrease. Note that the PSDs of *ζ*_5_ and *ζ*_6_ are 0 for *P* = 0.
(0.06 MB PDF)

Figure S4 Qualitative behavior of the system is insensitive to the parameter values. The base values of *k_A_*, *k_R_*, *γA_i_*, *γA_e_*, *γR*, *γC*, *k_C1_*, and *k_C2_* are individually decreased or increased by 10-fold (*k_A_* is only increased) and the dependence of noise in *C* (*η*_T_^2^: (A), *η*_E_^2^: (B) and *η*_I_^2^: (C)) on *P* and *γR* is examined.
(0.21 MB PDF)

Figure S5 The dependence of (A) *X_A_*+*X_R_*+*X_C_*, (B) *Y_AR_*, (C) *Y_AC_*, and (D) *Y_RC_* on *P* and *γR*. As defined in Equation 10 (Text S1), *X_A_*+*X_R_*+*X_C_* represents the contribution of extrinsic noise sources as independent entities and determines the basal dependence of *η*_E_^2^ on the parameters. *Ymm′* represents the contribution of correlation between two extrinsic noise sources, *ξm* and *ξm′*. The base parameter set (Table S2) is used for calculation.
(0.09 MB PDF)

Figure S6 Cell-cell variability affects population fitness. Population fitness is calculated by Monte Carlo simulation with different levels of cell-cell variability (*σ*). Parameter values are and *n* = 10,000, *μ* = 1, *λ* = 0.2, *ε* = 0.02, *M* = 1.8, and *F*_0_ = 1. Note that when *X_i_*≥*M* or *F_i_*<0, we set *F_i_* = 0.
(0.02 MB PDF)

Figure S7 Representative results of noise and PSDs of *C* calculated from numerical simulations (Equation 1). Time series of *C* is obtained over a time span of 400,000 min with sampling frequency of 10 min^−1^. Numerical simulation is implemented as in Figure 2. (A) The square of the total noise in *C* (*η*_T_^2^) is calculated from the time series (red dots). The blue line indicates *η*_T_^2^ calculated by the analytical approach. For these calculations, *γR* = 0.2 min^−1^. (B) The PSDs are calculated by taking absolute values of fast Fourier transformation of the time series. For these calculations, *P* = 2×10^−13^ L min^−1^, *γR* = 0.02 (blue), 0.2 (green), or 2 min^−1^ (red). The black lines indicate PSD calculated by the analytical approach.
(0.06 MB PDF)

Figure S8 Simulated histograms of *C* using Equation 1 for varying *P* and *γR*. A red line indicates the steady-state value of *C* calculated by the deterministic version of Equation 1, whereas a yellow line indicates the mean value of the corresponding distribution. Numerical simulation is implemented as in Figure 2.
(0.52 MB PDF)

Table S1 Noise sources and corresponding reactions.
(0.03 MB PDF)

Table S2 Base parameter values.
(0.05 MB PDF)
