# Supplementary material for: Noise Reduction by Diffusional Dissipation in a Minimal Quorum Sensing Motif
Source: PLoS Comput Biol. 2008 Aug 29;4(8):e1000167. doi: 10.1371/journal.pcbi.1000167 (PMC2507755; doi:10.1371/journal.pcbi.1000167)
Supplement: Text S1 — (0.23 MB PDF) [file pcbi.1000167.s001.pdf]

Supporting Information for

Noise reduction by diffusional dissipation in a minimal quorum sensing motif

Tanouchi et al.

1. Noise calculation

We use a Langevin approach to study noise. We first solve the system of equations linearized around the steady state in the frequency domain, and then calculate the PSD (power spectrum density) of each species. The energy theorem enables us to calculate the variance of each species in the temporal domain by integrating the PSDs in the frequency domain.

1.1 Linearization

Langevin equations for the systems are:

$$\begin{aligned}
\frac{dA_i}{dt} &= k_A - \gamma_{A_i} A_i - k_{C1} A_i R + k_{C2} C - P\left(\frac{A_i}{V_i} - \frac{A_e}{V_e}\right) \\
&\qquad + \zeta_1 + \zeta_2 + \zeta_3 + \zeta_4 + \zeta_5 + \xi_{A_i}, \\
\frac{dA_e}{dt} &= -\gamma_{A_e} A_e - P\left(\frac{A_e}{V_e} - \frac{A_i}{V_i}\right) - \zeta_5 + \zeta_6, \\
\frac{dR}{dt} &= k_R - \gamma_R R - k_{C1} A_i R + k_{C2} C + \zeta_3 + \zeta_4 + \zeta_7 + \zeta_8 + \xi_R, \\
\frac{dC}{dt} &= -\gamma_C C + k_{C1} A_i R - k_{C2} C - \zeta_3 - \zeta_4 + \zeta_9 + \xi_C.
\end{aligned} \tag{1}$$

The random timing and discrete nature of chemical reactions generate intrinsic noise sources. ζ_ℓ represents each intrinsic noise source associated with a reaction ℓ (Table S1). ξ_m (m = A_i, R and C)is the extrinsic noise source in each species. To be consistent with the main text we have,

$$\begin{aligned}
\sum_\ell \zeta_{A_i,\ell} &= \zeta_1 + \zeta_2 + \zeta_3 + \zeta_4 + \zeta_5, \\
\sum_\ell \zeta_{A_e,\ell} &= -\zeta_5 + \zeta_6, \\
\sum_\ell \zeta_{R,\ell} &= \zeta_3 + \zeta_4 + \zeta_7 + \zeta_8, \\
\sum_\ell \zeta_{C,\ell} &= -\zeta_3 - \zeta_4 + \zeta_9.
\end{aligned}$$

Excluding extrinsic noise terms, our model formulation is equivalent to the standard chemical Langevin equation [1].

The system is monostable. We denote the steady state values of A_i, A_e, R and C as A_i^{ss}, A_e^{ss}, R^{ss} and C^{ss}, respectively. We linearize the model Eq. (1) around the steady states by using

$$
\begin{aligned}
A_i &= A_i^{ss} + \Delta A_i, \\
A_e &= A_e^{ss} + \Delta A_e, \\
R &= R^{ss} + \Delta R, \\
C &= C^{ss} + \Delta C.
\end{aligned}
$$

At the steady state, each noise process can be considered as a stationary process. The linearization gives

$$
\begin{aligned}
\frac{d\Delta A_i}{dt} &= -\gamma_{A_i}\Delta A_i - k_{C1}R^{ss}\Delta A_i - k_{C1}A_i^{ss}\Delta R + k_{C2}\Delta C - P\left(\frac{\Delta A_i}{V_i} - \frac{\Delta A_e}{V_e}\right) \\
&\qquad + \zeta_1 + \zeta_2 + \zeta_3 + \zeta_4 + \zeta_5 + \xi_{A_i}, \\
\frac{d\Delta A_e}{dt} &= -\gamma_{A_e}\Delta A_e - P\left(\frac{\Delta A_e}{V_e} - \frac{\Delta A_i}{V_i}\right) - \zeta_5 + \zeta_6, \qquad (2) \\
\frac{d\Delta R}{dt} &= -\gamma_R\Delta R - k_{C1}R^{ss}\Delta A_i - k_{C1}A_i^{ss}\Delta R + k_{C2}\Delta C + \zeta_3 + \zeta_4 + \zeta_7 + \zeta_8 + \xi_R, \\
\frac{d\Delta C}{dt} &= -\gamma_C\Delta C + k_{C1}R^{ss}\Delta A_i + k_{C1}A_i^{ss}\Delta R - k_{C2}\Delta C - \zeta_3 - \zeta_4 + \zeta_9 + \xi_C.
\end{aligned}
$$

1.2 PSD calculation

Here, we focus on solving noise for the complex (C). The same scheme applies to other species as well. Suppose the noise process is observed over the interval $[-T/2, T/2]$ and its Fourier transformation of Eq. (2) exists. Taking the Fourier transformation and solving for ΔC give;

$$
\Delta\hat{C}(f) = \sum_{\ell} H_{\ell}^{\zeta}(f)\hat{\zeta}_{\ell}(f) + \sum_{m} H_m^{\xi}(f)\hat{\xi}_m(f),
$$

where hats denote variables evaluated in the frequency domain (f represents ordinary frequency); H_{ℓ}^{ζ} and H_m^{ξ} are frequency domain transfer functions for intrinsic and extrinsic noise sources.

Unilateral PSD of ΔC ($S_{\Delta C}$) can be calculated as

$$
\begin{aligned}
S_{\Delta C}(f) &= \lim_{T\to\infty} \frac{2\overline{\left|\Delta\hat{C}\right|^2}}{T} \\
&= \lim_{T\to\infty} \frac{2\overline{\left|\sum_\ell H_\ell^\zeta(f)\hat{\zeta}_\ell + \sum_m H_m^\xi(f)\hat{\xi}_m\right|^2}}{T},
\end{aligned}
\tag{3}
$$

where bars denote ensemble average. The statistical properties of intrinsic noise sources are:

$$
\begin{aligned}
&\langle \zeta_k \rangle = 0, \\
&\langle \zeta_k(t)\zeta_{k'}(t+\tau) \rangle = v_k \delta(\tau)\delta_{k,k'} \qquad (k = 1,\ldots,9),
\end{aligned}
\tag{4}
$$

where v_k is an average reaction rate of the corresponding reaction at the steady state as summarized in the Table below, $\delta(\tau)$ is the Dirac's delta function, and $\delta_{k,k'}$ is the Kronecker's delta function.

Noise source	Reaction	Average reaction rate (v_k)
ζ_1	AHL production	k_A
ζ_2	Internal AHL (A_i) decay	$\gamma_{A_i} A_i^{ss}$
ζ_3	Complex association	$k_{C1} A_i^{ss} R^{ss}$
ζ_4	Complex dissociation	$k_{C2} C^{ss}$ $k_{C2} C^{ss}$
ζ_5	Diffusion	$P\left\|A_i^{ss}/V_i - A_e^{ss}/V_e\right\|$
ζ_6	External AHL (A_e) decay	$\gamma_{A_e} A_e^{ss}$
ζ_7	LuxR production	k_R
ζ_8	LuxR decay	$\gamma_R R^{ss}$
ζ_9	Complex decay	$\gamma_C C^{ss}$

In the base model, we assume that all extrinsic noise sources are fully correlated ($\xi_{A_i}/\alpha_{A_i} = \xi_R/\alpha_R = \xi_C/\alpha_C = \xi$, where α_m determines the relative strength of each extrinsic noise sources), and the spectrum is white. Then, we have

$$
\begin{aligned}
&\langle \xi \rangle = 0, \\
&\langle \xi(t)\xi(t+\tau) \rangle = \beta\delta(\tau),
\end{aligned}
\tag{5}
$$

where β is the effective magnitude of the extrinsic noise source. Under these conditions (Eqs. (4) and (5)), Eq. (3) becomes:

$$
\begin{aligned}
S_{\Delta C} &= \lim_{T\to\infty} \frac{2\overline{\left|\sum_\ell H_\ell^\zeta \hat{\zeta}_\ell + \hat{\xi}\sum_m \alpha_m H_m^\xi\right|^2}}{T} \\
&= \lim_{T\to\infty} 2\left\{\sum_\ell \left|H_\ell^\zeta\right|^2 \frac{\overline{\left|\hat{\zeta}_\ell\right|^2}}{T} + \frac{\overline{\left|\hat{\xi}\right|^2}}{T}\left|\sum_m \alpha_m H_m^\xi\right|^2\right\} \\
&= \sum_\ell \left|H_\ell^\zeta\right|^2 S_\ell^\zeta + S^\xi \left|\sum_m \alpha_m H_m^\xi\right|^2,
\end{aligned} \tag{6}
$$

where S_ℓ^ζ and S^ξ denote PSD of intrinsic and extrinsic noise sources.

Eqs. (4) and (5) lead to $S_\ell^\zeta = 2v_\ell$ and $S^\xi = 2\beta$. If all extrinsic noise sources have the same magnitude ($\alpha_{A_i} = \alpha_R = \alpha_C = 1$), we have

$$
\begin{aligned}
S_{\Delta C} &= \sum_\ell \left|H_\ell^\zeta\right|^2 S_\ell^\zeta + S^\xi \left|\sum_m H_m^\xi\right|^2 \\
&= \sum_\ell \left|H_\ell^\zeta\right|^2 S_\ell^\zeta + \left|H^\xi\right|^2 S^\xi,
\end{aligned} \tag{7}
$$

where $H^\xi = \sum_m H_m^\xi$.

1.3 Noise calculation by the energy theorem

For the gated noise processes $x(t)$ (noise processes observed over the finite time interval $[-T/2, T/2]$, where $x(t) = 0$ outside of this interval), the energy theorem states that

$$
\lim_{T\to\infty} \frac{1}{T}\int_{-\infty}^{\infty} x(t)^2\,dt = \lim_{T\to\infty} \int_0^\infty \frac{2\left|\hat{x}(f)\right|^2}{T}\,df \ . \tag{8}
$$

The right hand side uses the relationship: $\int_{-\infty}^{\infty} \left|\hat{x}(f)\right|^2 df = \int_0^\infty 2\left|\hat{x}(f)\right|^2 df$. This is possible because, as $x(t)$ is a real process, the integrand is an even function. Taking the ensemble average of both sides of Eq. (8) gives

$$
\sigma_x^2 + \left(\overline{x(t)}\right)^2 = \int_0^\infty S_x(f)\,df \ , \tag{9}
$$

where σ_x^2 is the variance of $x(t)$.

By using Eq. (9), the variance of C is calculated as

$$\sigma_{\Delta C}^2 + \left(\overline{\Delta C(t)}\right)^2 = \sigma_C^2 = \int_0^\infty S_{\Delta C}(f)df \ .$$

Also, we can see from Eq. (6) that

$$\eta_T^2 = \eta_I^2 + \eta_E^2,$$
$$\eta_I^2 = \frac{1}{(C^{ss})^2}\int_0^\infty 2\sum_\ell \left|H_\ell^\zeta\right|^2 v_\ell df,$$
$$\eta_E^2 = \frac{1}{(C^{ss})^2}\int_0^\infty 2\beta\left|\sum_m \alpha_m H_m^\xi\right|^2 df,$$

where η_T^2, η_I^2 and η_E^2 are the total noise, intrinsic noise and extrinsic noise, respectively.

2. Impact of the dimerization of the complex

In this section, we incorporate the homodimerization of C into a dimer (D) and examine how the noise in D changes depending on different P and γ_R using the same analytical approach described above. The system now becomes

$$\frac{dA_i}{dt} = k_A - \gamma_{A_i}A_i - k_{C1}A_iR + k_{C2}C - P\left(\frac{A_i}{V_i} - \frac{A_e}{V_e}\right)$$
$$+\zeta_1 + \zeta_2 + \zeta_3 + \zeta_4 + \zeta_5 + \xi_{A_i},$$
$$\frac{dA_e}{dt} = -\gamma_{A_e}A_e - P\left(\frac{A_e}{V_e} - \frac{A_i}{V_i}\right) - \zeta_5 + \zeta_6,$$
$$\frac{dR}{dt} = k_R - \gamma_R R - k_{C1}A_iR + k_{C2}C + \zeta_3 + \zeta_4 + \zeta_7 + \zeta_8 + \xi_R,$$
$$\frac{dC}{dt} = -\gamma_C C + k_{C1}A_iR - k_{C2}C - 2k_{D1}C^2 + 2k_{D2}D$$
$$-\zeta_3 - \zeta_4 + \zeta_9 + 2\zeta_{10} + 2\zeta_{11} + \xi_C,$$
$$\frac{dD}{dt} = -\gamma_D D + k_{D1}C^2 - k_{D2}D - \zeta_{10} - \zeta_{11} + \zeta_{12} + \xi_D,$$

where k_{D1} and k_{D2} are association and dissociation rate constants of D; ζ_{10}, ζ_{11} and ζ_{12} are intrinsic noise sources originating from dimer association, dissociation and decay; and ξ_D is an extrinsic noise sources for D. We set k_{D1} and k_{D2} to be the same as k_{C1} and k_{C2}, respectively. We also assume that all extrinsic noise sources are correlated and of the same magnitudes. As shown in Figure S1, extrinsic noise in D and the gain of the transfer

function exhibit qualitatively the same dependence on P and γ_R as noise in C without dimerization (Figure 2 and 3).

3. Effects of population size on noise attenuation

Figure S2 shows numeric simulations of noise in A_i, A_e, R and C when the system contains multiple cells coupled by quorum sensing signal. Simulations are performed for the following conditions:

1) Each cell has its own microenvironment (e.g. no coupling).
2) 100 cells are divided into ten populations, each of which contains ten cells coupled to one another via a common environment.
3) 100 cells form 1 population of 100 coupled cells.

In each case, the cell density is kept constant. Essentially, case 1 has no coupling between cells and case 3 has the strongest. If the coupling plays a significant role in reducing cellular noise, we would expect the least variability in case 3 and the most in case 1.

However, numerical simulations indicate no significant change of noise in A_i, R and C in the population from 1 to 100 (<2 %). In contrast, noise in A_e is reduced by >80 % while its absolute value of noise is >100 fold smaller than A_i, R and C. In Figure S2, we use a diffusion rate constant of 2×10^{-13} L min^{-1} and the similar result is obtained with a slower diffusion rate constant of 2×10^{-15} L min^{-1} (data not shown). Thus, the noise reduction in A_e has little impact on noise in C. Also, as discussed in the main text, the fast turnover of the signal plays a major role in noise attenuation of C, and coupling of a cell with its extracellular environment but not with multiple cells is sufficient to achieve the fast signal turnover via diffusion. These results support that noise calculation in a single cell model is sufficient to examine the noise characteristics.

4. Intrinsic noise dependence

As mentioned in the main text, the total intrinsic noise does not change significantly when diffusion and R decay rates are modulated. However, individual indeed varies with these parameters, but they cancel out each other. Figure S3 shows how the PSD of each intrinsic noise source depends on diffusion rates. The same trend is observed when the R decay rate is varied (data not shown).

5. Parameters and sensitivity analysis

Parameters for the model are obtained from experimental measurements found in the literature or estimated based on the observed biological behavior of the system (Table S2). We also test if noise modulation of C by P and γ_R is sensitive to the choice of other

parameters, including k_A, k_R, γ_{A_i}, γ_{A_e}, γ_R, γ_C, k_{C1} and k_{C2}. Except for k_A, each of these parameters is decreased and increased by 10 fold while the other parameters are held constant. k_A is only increased by 10 fold because the steady state value of A_i becomes less than 1 when k_A is decreased by 10 fold.

As shown in Figure S4, in general, the total noise in C decreases as P or γ_R increases. Exceptions are when k_R is increased and γ_R is decreased (Figure S4A, $k_R \times 10$ and $\gamma_R/10$, respectively). In the $k_R \times 10$ case, when P is increased from 2×10^{-13} to 2×10^{-11} L min^{-1}, the total noise in C increases slightly. This increase is primarily due to the increase in the intrinsic noise (Figure S4C). In $k_R \times 10$ case, the intrinsic noise becomes comparable to extrinsic noise especially for fast diffusion (Figure S4B and C). However, we note that extrinsic noise consistently decreases with increasing P and the dynamic range of extrinsic noise is ~20 fold larger than that of intrinsic noise. This is also true for the $\gamma_R/10$ case. Therefore, we conclude that the qualitative behavior of extrinsic noise is insensitive to the base parameter sets, and as long as extrinsic noise dominates total noise, the behavior of total noise is also insensitive.

6. Various correlation of extrinsic noise sources

In the previous sections, we assumed that the extrinsic noise sources (ξ_{A_i}, ξ_R and ξ_C) are fully correlated with one another. This condition is not necessarily satisfied in a real biological system. Here, we consider the general case where the extrinsic noise sources are correlated to varying extent or not correlated.

Assuming that extrinsic noise sources are uncorrelated with intrinsic noise sources, PSD of extrinsic noise in C ($S_{\Delta C_E}$) can be written as

$$\begin{aligned} S_{\Delta C_E} &= \lim_{T\to\infty} \frac{2\overline{\left|\sum_m H_m^{\xi}\hat{\xi}_m\right|^2}}{T} \\ &= \lim_{T\to\infty} \frac{2}{T}\left\{\sum_m \left|H_m^{\xi}\right|^2 \overline{\left|\hat{\xi}_m\right|^2} + \sum_{m\neq m'} H_m^{\xi} H_{m'}^{\xi *} \overline{\hat{\xi}_m \hat{\xi}_{m'}^{*}}\right\} \\ &= \sum_m S_m^{\xi}\left|H_m^{\xi}\right|^2 + \sum_{m\neq m'} S_{mm'}^{\xi} H_m^{\xi} H_{m'}^{\xi *}, \end{aligned}$$

where $S_{mm'}^{\xi}$ ($m \neq m'$) is a cross-spectral density and $*$ denotes complex conjugate.

When extrinsic noise sources are correlated only at simultaneous time points (i.e. $\langle \xi_m(t)\xi_{m'}(t+\tau)\rangle = R_{mm'}\delta(\tau)$, where $R_{mm'}$ is a constant),

$$S_{\Delta C_E} = \sum_m S_m^\xi \left| H_m^\xi \right|^2 + \sum_{m \neq m'} S_{mm'}^\xi \Re[H_m^\xi H_{m'}^{\xi *}]$$
$$= 2\beta \sum_m \alpha_m^2 \left| H_m^\xi \right|^2 + 2\beta \sum_{m \neq m'} r_{mm'} \alpha_m \alpha_{m'} \Re[H_m^\xi H_{m'}^{\xi *}],$$

where $\Re[H]$ is a real part of H and

$$r_{mm'} = \frac{\langle \xi_m(t) \xi_{m'}(t) \rangle}{\langle \xi_m(t)^2 \rangle^{1/2} \langle \xi_{m'}(t)^2 \rangle^{1/2}}$$

is the correlation coefficient between ξ_m and $\xi_{m'}$ ($r_{mm'} = r_{m'm}$).

If all extrinsic noise sources have the same magnitude (i.e. $\alpha_m = 1$), we obtain

$$S_{\Delta C_E} = 2\beta \left(\sum\nolimits_m \left| H_m^\xi \right|^2 + \sum\nolimits_{m \neq m'} r_{mm'} \Re[H_m^\xi H_{m'}^{\xi *}] \right),$$
$$\eta_E^2 = \frac{2\beta}{(C^{ss})^2} \int_0^\infty \left(\sum\nolimits_m \left| H_m^\xi \right|^2 + \sum\nolimits_{m \neq m'} r_{mm'} \Re[H_m^\xi H_{m'}^{\xi *}] \right) df \tag{10}$$
$$= \frac{2\beta}{(C^{ss})^2} \left(X_A + X_R + X_C + r_{AR} Y_{AR} + r_{AC} Y_{AC} + r_{RC} Y_{RC} \right),$$

where $X_m = \int_0^\infty \left| H_m^\xi \right|^2 df$ and $Y_{mm'} = \int_0^\infty 2\Re[H_m^\xi H_{m'}^{\xi *}] df$ (note $\Re[H_m^\xi H_{m'}^{\xi *}] = \Re[H_{m'}^\xi H_m^{\xi *}]$). As $2\beta/(C^{ss})^2$ is constant, the dependence of X_m and $Y_{mm'}$ on P and γ_R directly reflects the dependence of η_E^2. The term $X_A + X_R + X_C$ represents the contribution of extrinsic noise sources as independent entities and reflects a basal dependence of η_E^2. $Y_{mm'}$ represents the contribution of correlation between two extrinsic noise sources, ξ_m and $\xi_{m'}$.

The combination of r_{AR}, r_{AC} and r_{RC} has a constraint (in addition to $-1 \le r_{mm'} \le 1$) and cannot be completely arbitrary. Partial correlation of ξ_i and ξ_j given ξ_k ($r_{ij \cdot k}$) is

$$r_{ij \cdot k} = \frac{r_{ij} - r_{ik} r_{jk}}{\sqrt{(1 - r_{ik}^2)(1 - r_{jk}^2)}}.$$

Since $-1 \le r_{ij \cdot k} \le 1$, we have

$$\left(\frac{r_{ij} - r_{ik}r_{jk}}{\sqrt{(1-r_{ik}^2)(1-r_{jk}^2)}}\right)^2 \le 1,$$
$$r_{ij}^2 - 2r_{ij}r_{ik}r_{jk} + r_{ik}^2 r_{jk}^2 \le 1 - r_{ik}^2 - r_{jk}^2 + r_{ik}^2 r_{jk}^2,$$
$$r_{ij}^2 - (2r_{ik}r_{jk})r_{ij} - (1 - r_{ik}^2 - r_{jk}^2) \le 0.$$

Thus, we obtain the range of r_{ij} as a function of r_{ik} and r_{jk},

$$\text{Max}[-1, r_{ik}r_{jk} - \sqrt{(1-r_{ik}^2)(1-r_{jk}^2)}] \le r_{ij} \le \text{Min}[1, r_{ik}r_{jk} + \sqrt{(1-r_{ik}^2)(1-r_{jk}^2)}].$$

6.1 Uncorrelated Extrinsic Noise Sources

When extrinsic noise sources are uncorrelated (i.e. $r_{AR} = r_{AC} = r_{RC} = 0$), only the $X_A + X_R + X_C$ term (Eq. (10)) matters. As shown in Figure S5A, $X_A + X_R + X_C$ shows qualitatively the same dependence as the case with fully correlated extrinsic noise sources (i.e. $r_{AR} = r_{AC} = r_{RC} = 1$). It decreases with increasing *P* and γ_R.

6.2 Arbitrarily Correlated Extrinsic Noise Sources

If extrinsic noise sources are correlated, $Y_{mm'}$'s terms contribute to η_E^2. Figure S5B–D show $Y_{mm'}$ as a function of *P* and γ_R calculated with base parameter values (Table S2), as also summarized below:

Direction	Y_{AR}	Y_{AC}	Y_{RC}
Increasing *P*	Monotonic decrease	Monotonic decrease	Monotonic increase or decrease depending on γ_R
Increasing γ_R	Non-monotonic change	Monotonic increase	Monotonic decrease

We note that the dependence of Y_{AR} and Y_{AC} on γ_R and the dependence of Y_{RC} on *P* are negligible in comparison to that of $X_A + X_R + X_C$ on these parameters. Thus, they do not affect the overall dependence of η_E^2 on γ_R and *P*.

Since the dependence of Y_{AR} and Y_{AC} on *P* and that of Y_{RC} on γ_R have the same trend as $X_A + X_R + X_C$, we expect η_E^2 to monotonically decrease with increasing *P* and γ_R if extrinsic noise sources are all positively correlated ($r_{mm'}$'s > 0). In contrast, when $r_{mm'}$ values are negative, increasing *P* and γ_R may increase $r_{mm'}Y_{mm'}$. To systematically examine the effect of arbitrary correlations of extrinsic noise sources, we use the following metrics:

$$\pi_1 = \frac{\eta_E^2(P_{\max}, \gamma_{R\min})}{\eta_E^2(P_{\min}, \gamma_{R\min})},$$

$$\pi_2 = \frac{\eta_E^2(P_{\min}, \gamma_{R\max})}{\eta_E^2(P_{\min}, \gamma_{R\min})},$$

$$\pi_3 = \frac{\eta_E^2(P_{\max}, \gamma_{R\max})}{\eta_E^2(P_{\min}, \gamma_{R\min})},$$

where $\eta_E^2(P_{\min}, \gamma_{R\min})$ means η_E^2 evaluated at $P = P_{\min}$ and $\gamma_R = \gamma_{R\min}$, and $P_{\min}$ = 2×10^{-15} L min^{-1}, $P_{\max}$ = 2×10^{-11} L min^{-1}, $\gamma_{R\min}$ = 0.02 min^{-1} and $\gamma_{R\max}$ = 2 min^{-1}.

For ~95% of 10000 randomly generated combinations of $r_{mm'}$, π_3 is always less than π_1, π_2 and 1, indicating noise reduction by simultaneous increase in P and γ_R (π_1 and/or π_2 may become greater than 1 if $r_{mm'}$ is negative). Such noise reduction is always synergistic. By synergistic, we mean that the noise reduction by increased γ_R is enhanced by larger P and vice versa. We thus use $(1-\pi_3) > (1-\pi_1) + (1-\pi_2)$ to test synergy (i.e. noise reduction at ($P_{\max}, \gamma_{R\max}$) is larger than the sum of noise reduction at ($P_{\max}, \gamma_{R\min}$) and at ($P_{\min}, \gamma_{R\max}$)). In those cases that do not exhibit noise reduction, either r_{AC} or r_{RC} is close to -1. Given the possible global mechanisms contributing to extrinsic noise sources (fluctuation in degradation machinery, dilution by stochastic cell growth/division, for example), it is highly unlikely that extrinsic noise sources of A_i and C, or R and C are almost completely anti-correlated. Therefore, we expect the synergistic noise reduction for arbitrarily correlated extrinsic noise sources.

7. Impact of noise on population fitness

It has been shown that extrinsic noise tends to be slow fluctuations that persist over approximately one cell cycle [2]. Considering that many genes are controlled by QS [3], if the expression of QS-regulated genes (effectors) is costly, persisting noise in the gene expression might create significant cell-cell variations in the growth rate among the population. Here, we perform a simple analysis to examine how such cell-cell variations may affect the fitness of population, where a costly target gene is controlled by quorum sensing.

For simplicity, we assume that the expression level of effectors in each cell (X_i ($i = 1, \ldots, n$)) follows a normal distribution (e.g. $X_i \sim N(\mu, \sigma^2)$), where n is the number of cells in the population. The expression level is assumed to sustain over one cell cycle. The cost of expressing the effectors in each cell (c_i) is described as $c_i = f_c(X_i)$, while the benefit (b_i) from the effectors can be described as $b_i = f_b(\overline{X}_n)$, where $\overline{X}_n$ is the

sample average of the expression level. We further assume that fitness of each cell (F_i) is $F_i = F_0 + b_i - c_i$, where F_0 is basal fitness which represents fitness when the expression of effectors is not triggered yet. For f_b and f_c, as used in a previous study [4], we employed

$$f_b(x) = \lambda x,$$

$$f_c(x) = \varepsilon \frac{x}{1 - x/M}.$$

We define population fitness as $\overline{F}_n = \frac{1}{n}\sum_i^n F_i$ which is calculated by randomly generating X's. To examine the effect of cell-cell variation in the expression level of effectors, we calculate the population fitness of 10000 cells with different σ. As shown in Figure S6, population fitness decreases as σ increases. This makes intuitive sense because the cost increases more than linear when the effectors level is increased and thus, population cost ($\frac{1}{n}\sum_i^n c_i$) increases as σ increases. On the other hand, population benefit ($\frac{1}{n}\sum_i^n b_i$) hardly changes as $\overline{X}_n$ is independent of σ (it changes only due to a sampling effect).

8. Model simplification

The DC component of the transfer function of extrinsic noise source, $\left|H^{\xi}(f = 0)\right|$ (Eq. (7)), is

$$\frac{(D_e\gamma_{A_i} + (D_i + \gamma_{A_i})\gamma_{A_e})(\gamma_R + 2A_i^{ss}k_{C1}) + 2(D_e + \gamma_{A_e})\gamma_R k_{C1} R^{ss}}{(D_e\gamma_{A_i} + (D_i + \gamma_{A_i})\gamma_{A_e})(\gamma_C(\gamma_R + A_i^{ss}k_{C1}) + \gamma_R k_{C2}) + (D_e + \gamma_{A_e})\gamma_C\gamma_R k_{C1} R^{ss}}, \quad (11)$$

where $D_i = P/V_i$ and $D_e = P/V_e$. If P is sufficiently small so that $D_e \ll \gamma_{A_e}$ (and therefore, $D_e + \gamma_{A_e} \approx \gamma_{A_e}$) is satisfied, Eq. (11) becomes

$$\frac{\gamma'_A(\gamma_R + 2A_i^{ss}k_{C1}) + 2\gamma_R k_{C1} R^{ss}}{\gamma'_A(\gamma_C(\gamma_R + A_i^{ss}k_{C1}) + \gamma_R k_{C2}) + \gamma_C\gamma_R k_{C1} R^{ss}}, \quad (12)$$

where $\gamma'_A = D_i + \gamma_{A_i}$.

Eq. (12) is, in fact, equivalent to the expression derived from the simplified system in Figure 4A (in the simplified system, A_i becomes A). Thus, the parameter dependence of the DC component as well as low frequency components in which Eq. (11) dominates the frequency response can be well captured by the simplified system at

least under the aforementioned condition. The same simplification is applied for the case with arbitrarily correlated extrinsic noise sources (not shown).

Also, if we differentiate Eq. (12) ($= f_1(\gamma'_A, \gamma_R)$) with respect to γ'_A and γ_R, we can show:

$$\frac{\partial f_1}{\partial \gamma'_A} = -\frac{R^{ss}\gamma_R^2 k_{C1}(\gamma_C + 2k_{C2})}{(\gamma'_A(\gamma_C(\gamma_R + A_i^{ss}k_{C1}) + \gamma_R k_{C2}) + \gamma_C\gamma_R k_{C1}R^{ss})^2} < 0,$$
$$\frac{\partial f_1}{\partial \gamma_R} = -\frac{A_i^{ss}\gamma_A'^2 k_{C1}(\gamma_C + 2k_{C2})}{(\gamma'_A(\gamma_C(\gamma_R + A_i^{ss}k_{C1}) + \gamma_R k_{C2}) + \gamma_C\gamma_R k_{C1}R^{ss})^2} < 0.$$

This result shows that the DC component and low frequency component whose behavior is represented by Eq. (12) monotonically decrease by fast turnover of *A* and *R*. In fact, we can show the similar inequalities for Eq. (11) ($= f_2(P, \gamma_R)$) that $\partial f_2/\partial P$, $\partial f_2/\partial \gamma_R < 0$ (not shown).

9. Comparison of analytical and numeric results

The total noise in *C* and its PSD are calculated by numerical simulations of Eq. (1) using base parameter sets with varying *P* ($0 - 2\times10^{-11}$ L min^{-1}) and γ_R (0.02 – 2 min^{-1}). Overall, our analytical approach shows excellent match with the simulation results; representative cases are shown in Figure S7. There is a significant deviation (e.g. η_T^2 = 0.46 and 0.24 for analytical approach and simulation, respectively in Figure S7A) when *P* = 0 L min^{-1} (or when *P* is close to 0). In this case, the distribution of *C* becomes widely spread and skewed (Figure S8); the linearization is no longer a good approximation. The skewed distribution of *C* is probably due to small number of molecules and nonlinearity of the system, and results in the discrepancy between its deterministic steady-state level and average level. When noise is small enough (e.g. the distribution is tight enough), the distribution is almost symmetric, and the deterministic steady state level and the average level show a good match.

While it has been shown that a bimolecular reaction can feature resonance/band-pass filtering effects [5] and that a monostable system (in deterministic domain) can exhibit noise-induced multistability [6], we did not observe such phenomena in our system.

References

1. Gillespie DT (2000) The chemical Langevin equation. Journal of Chemical Physics 113: 297.
2. Rosenfeld N, Young JW, Alon U, Swain PS, Elowitz MB (2005) Gene regulation at the single-cell level. Science 307: 1962--1965.
3. Schuster M, Lostroh CP, Ogi T, Greenberg EP (2003) Identification, timing, and signal specificity of Pseudomonas aeruginosa quorum-controlled genes: a transcriptome analysis. J Bacteriol 185: 2066--2079.
4. Dekel E, Alon U (2005) Optimality and evolutionary tuning of the expression level of a protein. Nature 436: 588--592.
5. Samoilov M, Arkin A, Ross J (2002) Signal processing by simple chemical systems. J Phys Chem A. pp. 10205-10221.
6. Samoilov M, Plyasunov S, Arkin AP (2005) Stochastic amplification and signaling in enzymatic futile cycles through noise-induced bistability with oscillations. Proc Natl Acad Sci U S A 102: 2310-2315.
