# Supplementary figures and images for: Noise Reduction by Diffusional Dissipation in a Minimal Quorum Sensing Motif
Source: PLoS Comput Biol. 2008 Aug 29;4(8):e1000167. doi: 10.1371/journal.pcbi.1000167 (PMC2507755; doi:10.1371/journal.pcbi.1000167)

A

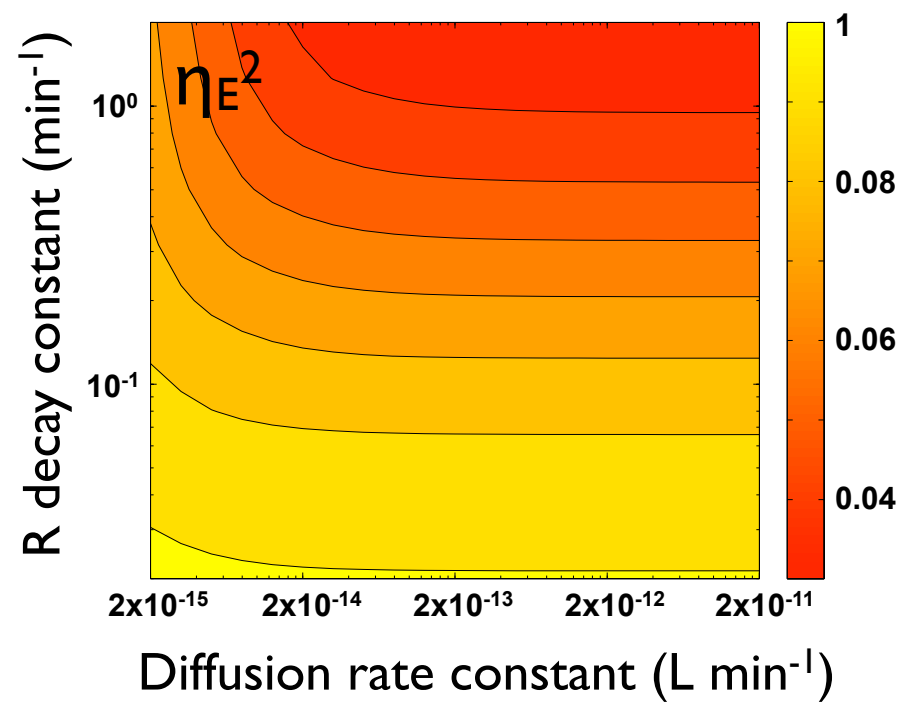

B

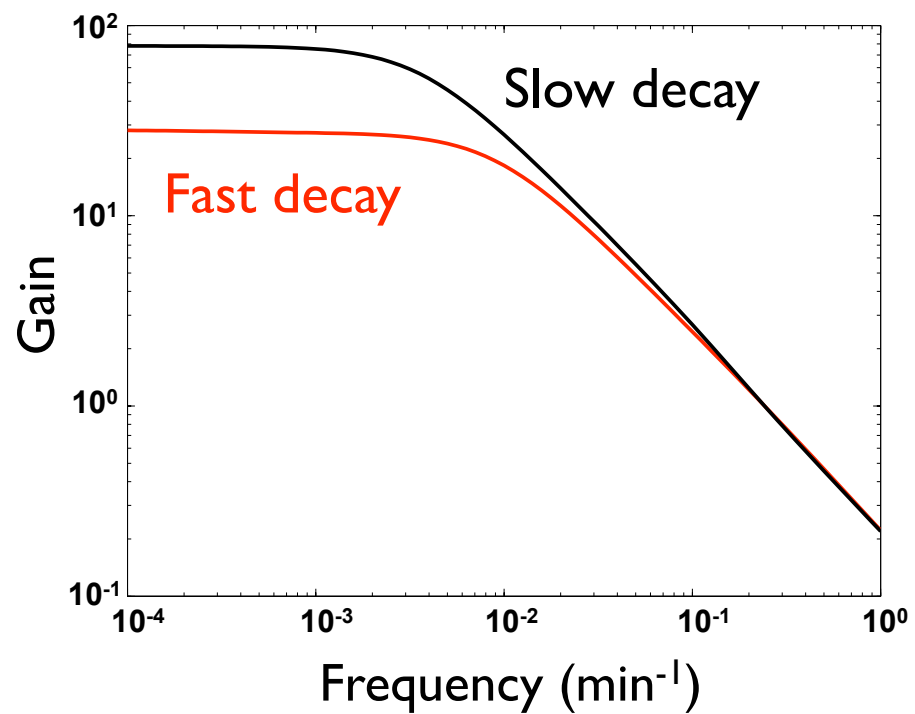

Supplement: Figure S1 — Impact of the dimerization reaction. Noise and PSD in D are calculated using the analytical approach with the base parameter set (Table S2). (A) Extrinsic noise in D for varying P and γR. Total noise in D shows the same dependence as the extrinsic noise is dominant (not shown). (B) The gain of extrinsic noise sources decreases as γR increases (from the black line (γR = 0.02 min−1) to the red line (γR = 2 min−1)). (0.06 MB PDF) [file pcbi.1000167.s002.pdf]

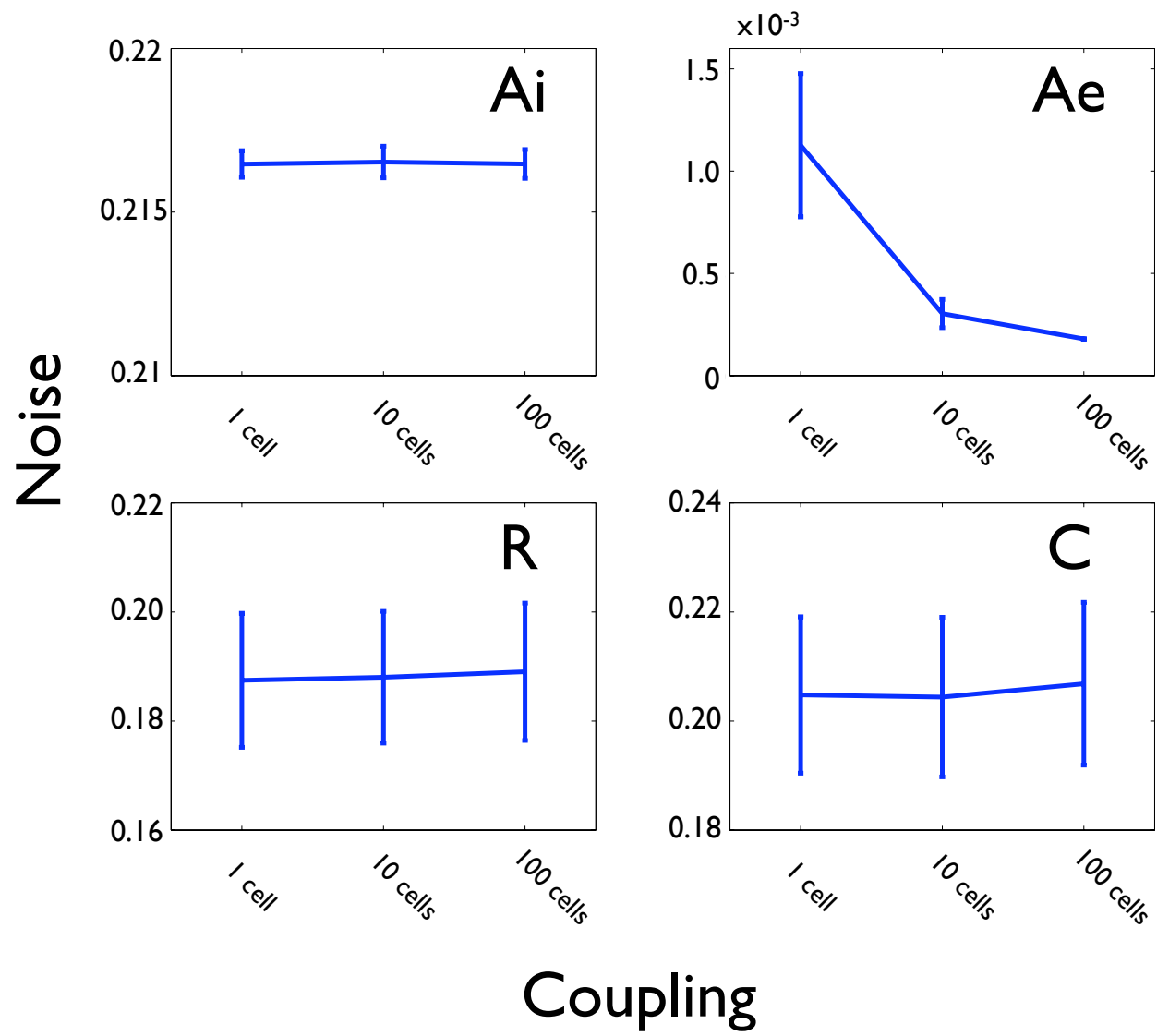

Supplement: Figure S2 — Simulation results of noise in A i, A e, R, and C under different coupling conditions: (1) each cell has its own microenvironment (e.g. no coupling). (2) 100 cells are divided into 10 populations each of which contains 10 cells coupled to one another via their environment, and (3) 100 cells form 1 population of 100 coupled cells. The conditions are indicated on the x-axis. The simulation is carried out as in Figure 2. Noise is calculated for individual cell from time course simulations for a span of 1,000 min (10,000 data points). Noise values shown are the average of 100 cells. For these calculations, P = 2×10^−13^ L min−1. (0.02 MB PDF) [file pcbi.1000167.s003.pdf]

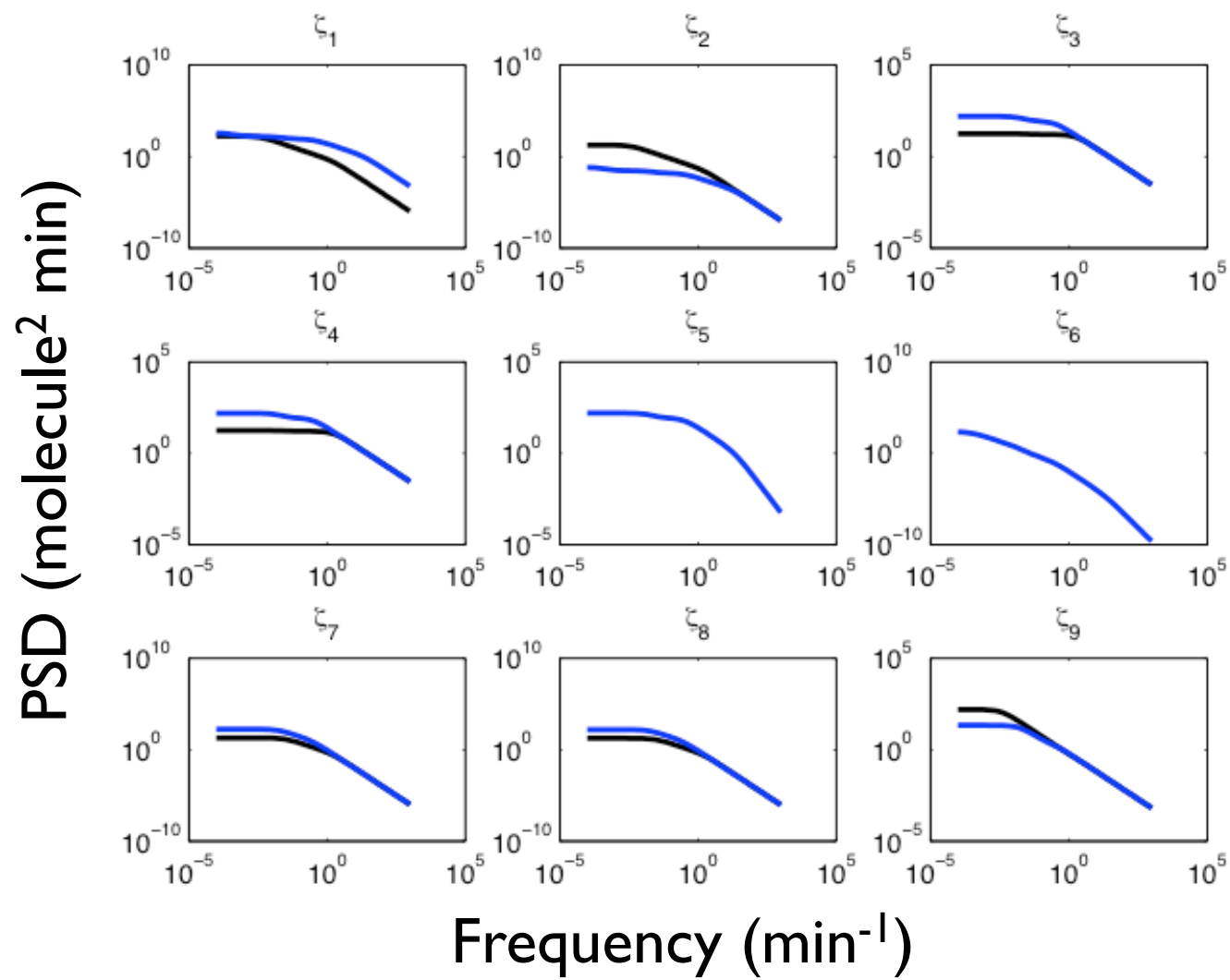

Supplement: Figure S3 — PSD of each intrinsic noise arising from the corresponding intrinsic noise source (ζ 1, ζ 2, …, ζ 9). P is changed from 0 (black line) to 2×10^−13^ L min−1 (blue line). Accordingly, the PSDs of ζ 1, ζ 3, ζ 4, ζ 7, and ζ 8 increase, while those of the other noise sources decrease. Note that the PSDs of ζ 5 and ζ 6 are 0 for P = 0. (0.06 MB PDF) [file pcbi.1000167.s004.pdf]

A

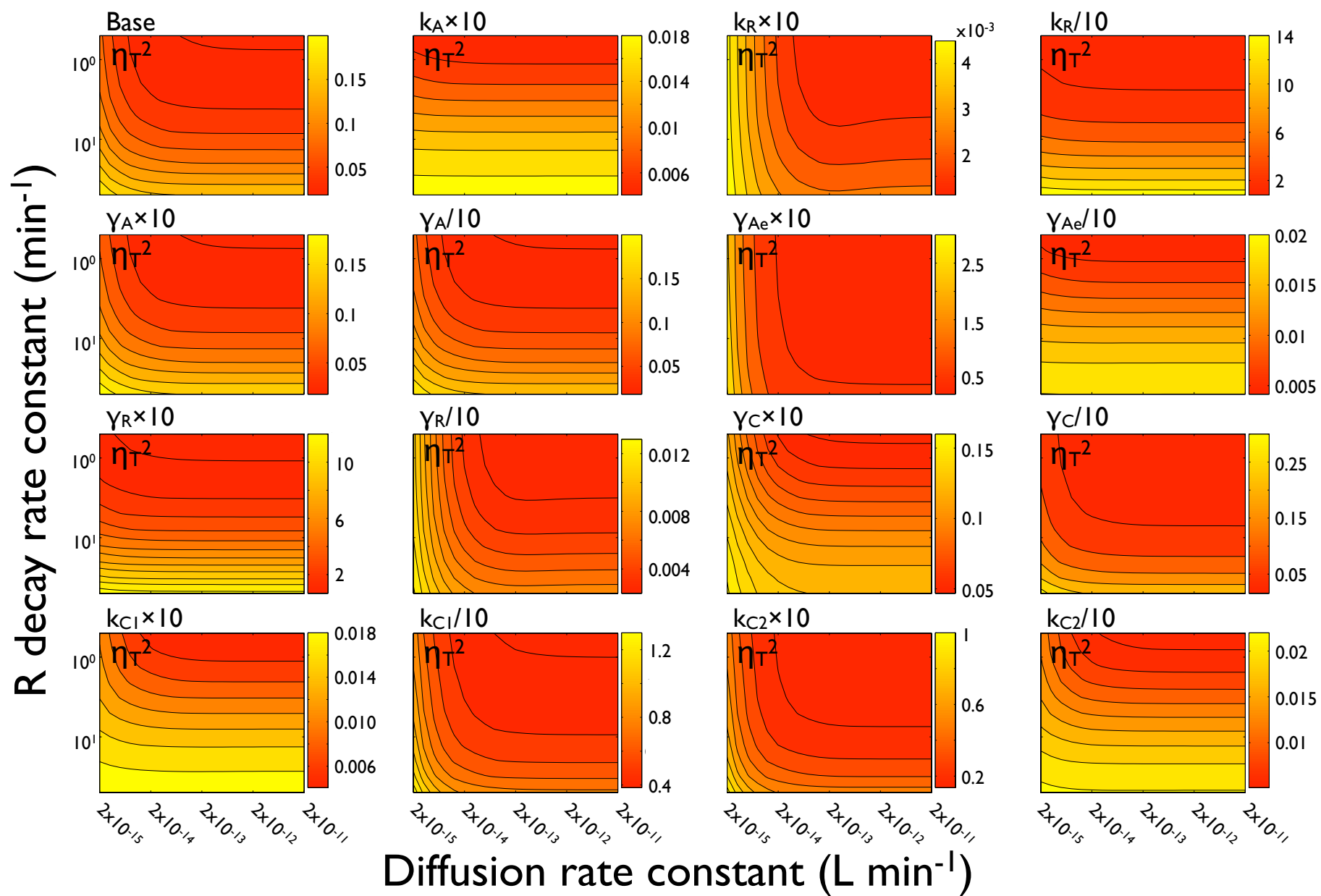

B

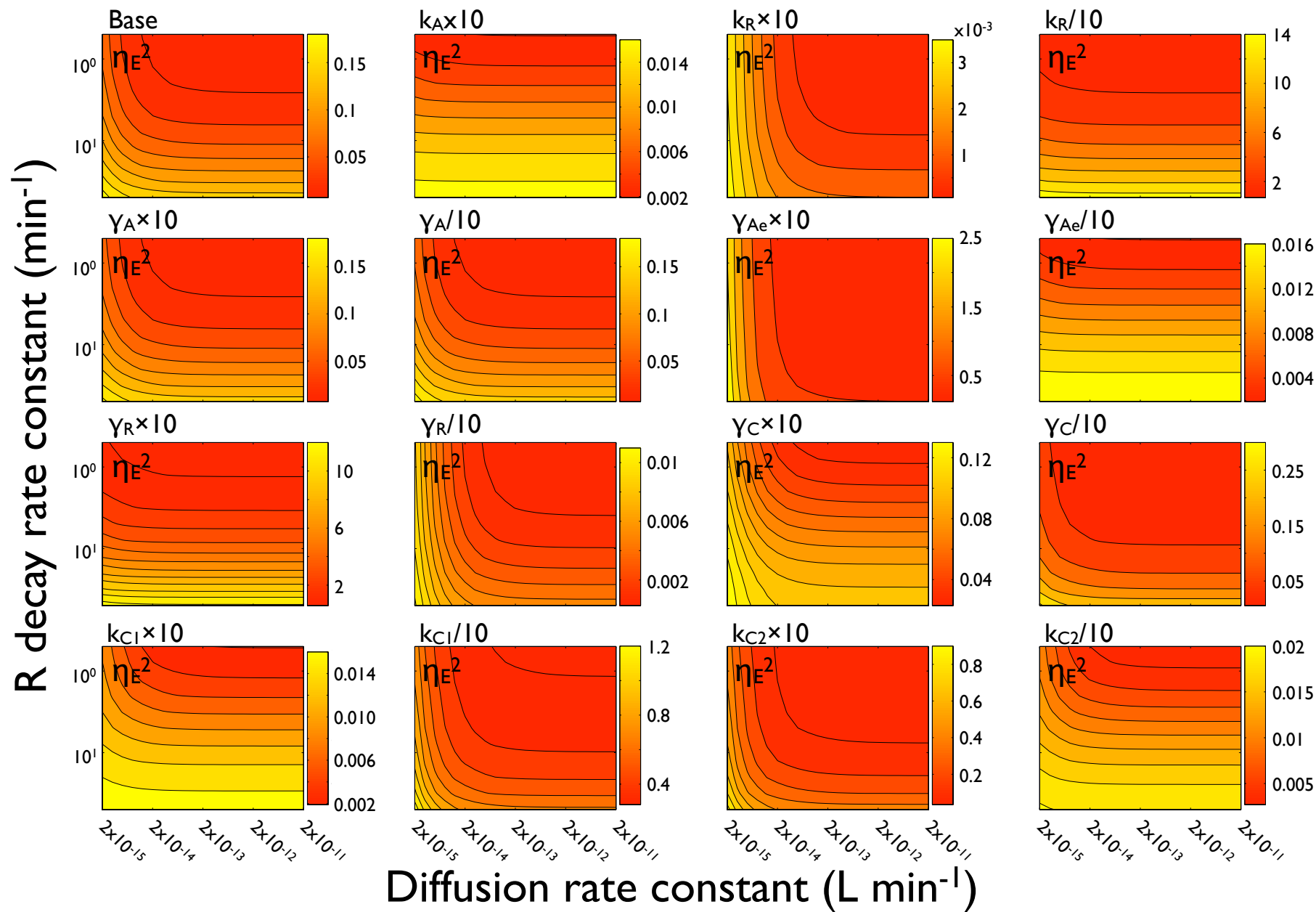

C

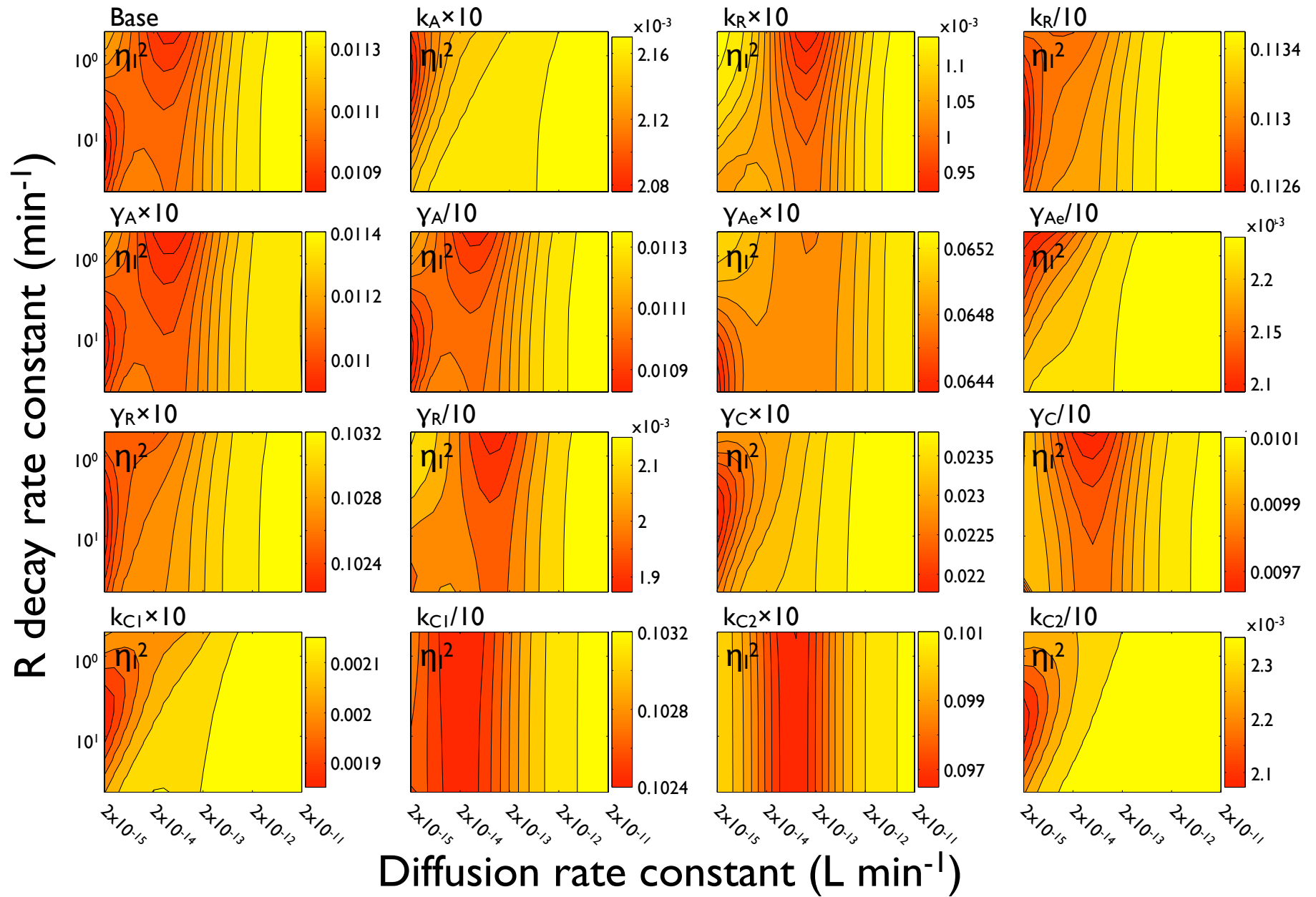

Supplement: Figure S4 — Qualitative behavior of the system is insensitive to the parameter values. The base values of kA, kR, γAi, γAe, γR, γC, kC1, and kC2 are individually decreased or increased by 10-fold (kA is only increased) and the dependence of noise in C (η T 2: (A), η E 2: (B) and η I 2: (C)) on P and γR is examined. (0.21 MB PDF) [file pcbi.1000167.s005.pdf]

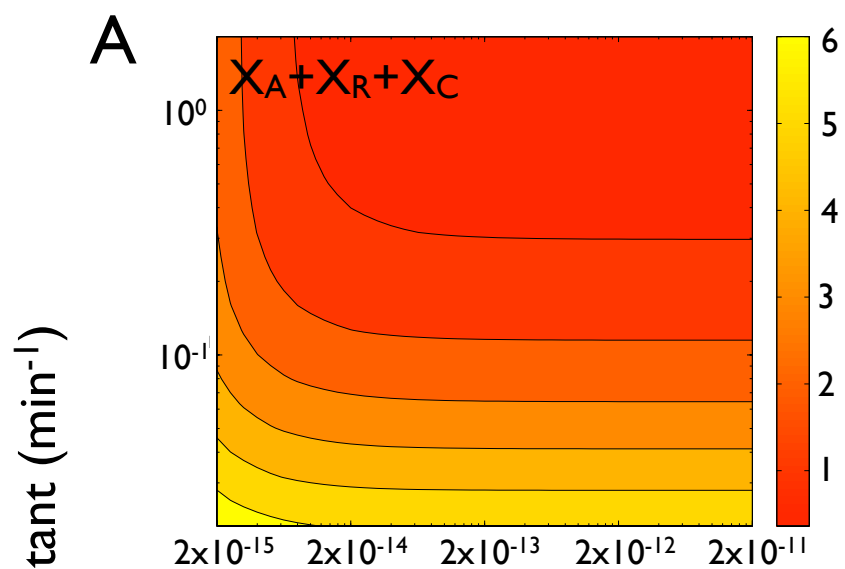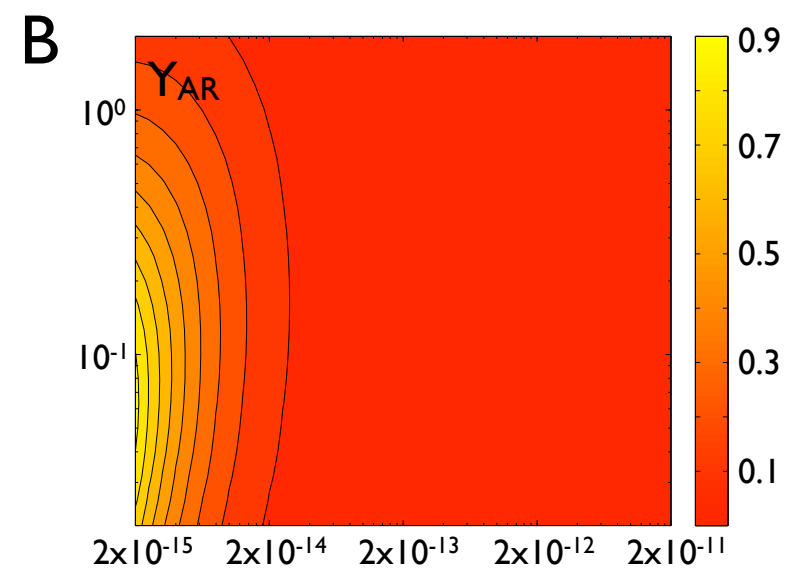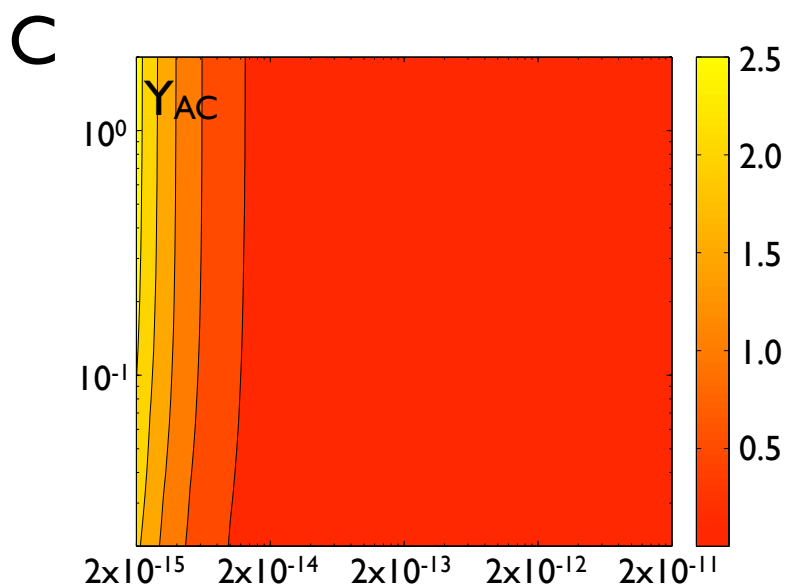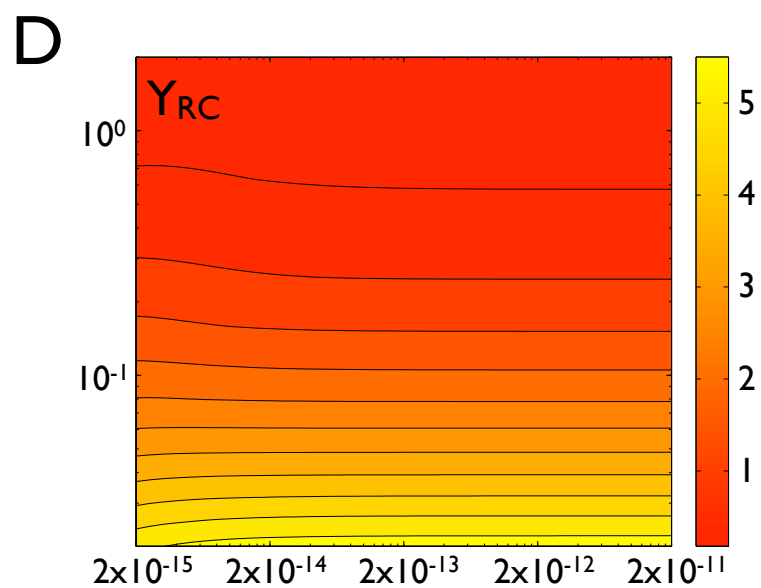

Diffusion rate constant ( $\text{L min}^{-1}$ )

Supplement: Figure S5 — The dependence of (A) XA+XR+XC, (B) YAR, (C) YAC, and (D) YRC on P and γR. As defined in Equation 10 (Text S1), XA+XR+XC represents the contribution of extrinsic noise sources as independent entities and determines the basal dependence of η E 2 on the parameters. Ymm′ represents the contribution of correlation between two extrinsic noise sources, ξm and ξm′. The base parameter set (Table S2) is used for calculation. (0.09 MB PDF) [file pcbi.1000167.s006.pdf]

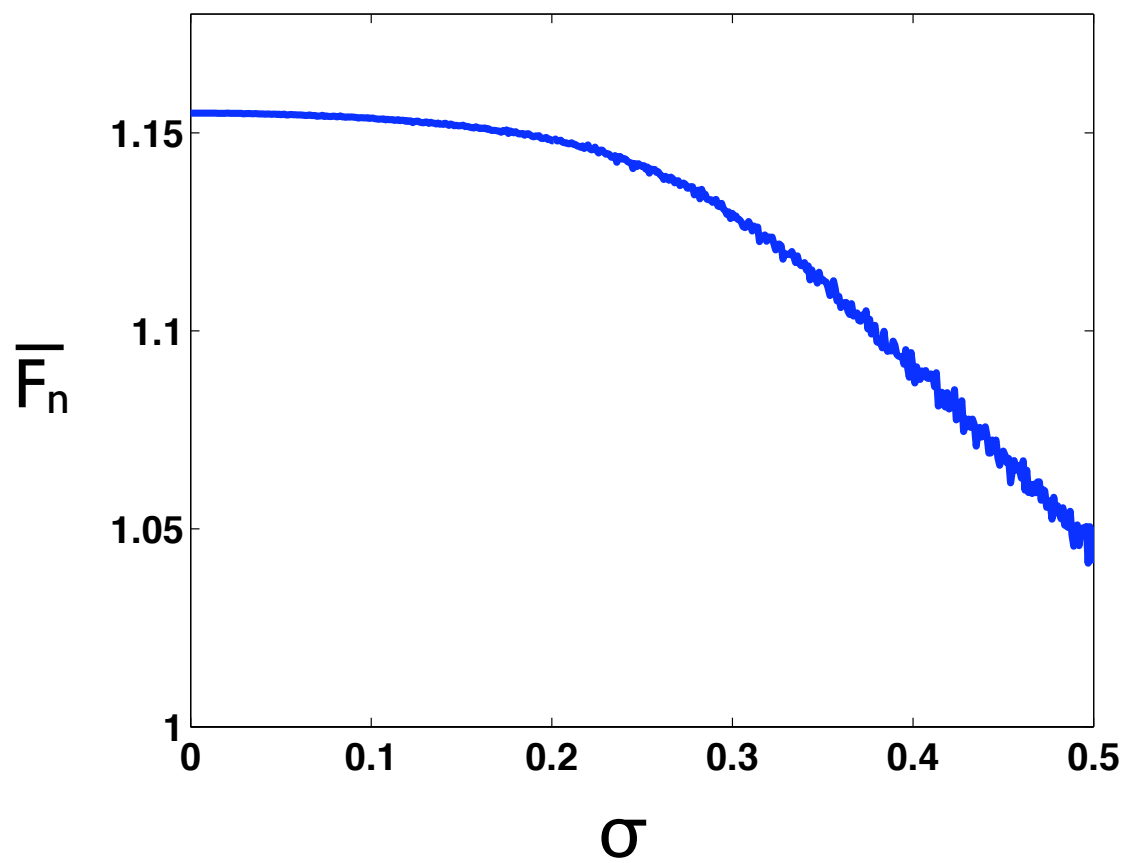

Supplement: Figure S6 — Cell-cell variability affects population fitness. Population fitness is calculated by Monte Carlo simulation with different levels of cell-cell variability (σ). Parameter values are and n = 10,000, μ = 1, λ = 0.2, ε = 0.02, M = 1.8, and F 0 = 1. Note that when Xi≥M or Fi<0, we set Fi = 0. (0.02 MB PDF) [file pcbi.1000167.s007.pdf]

**A**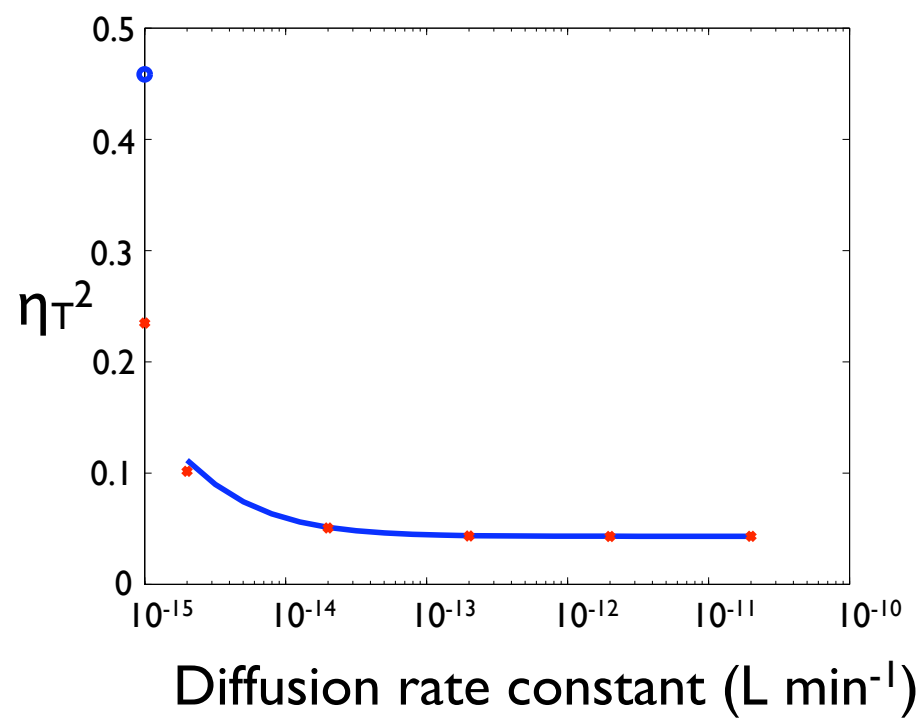**B**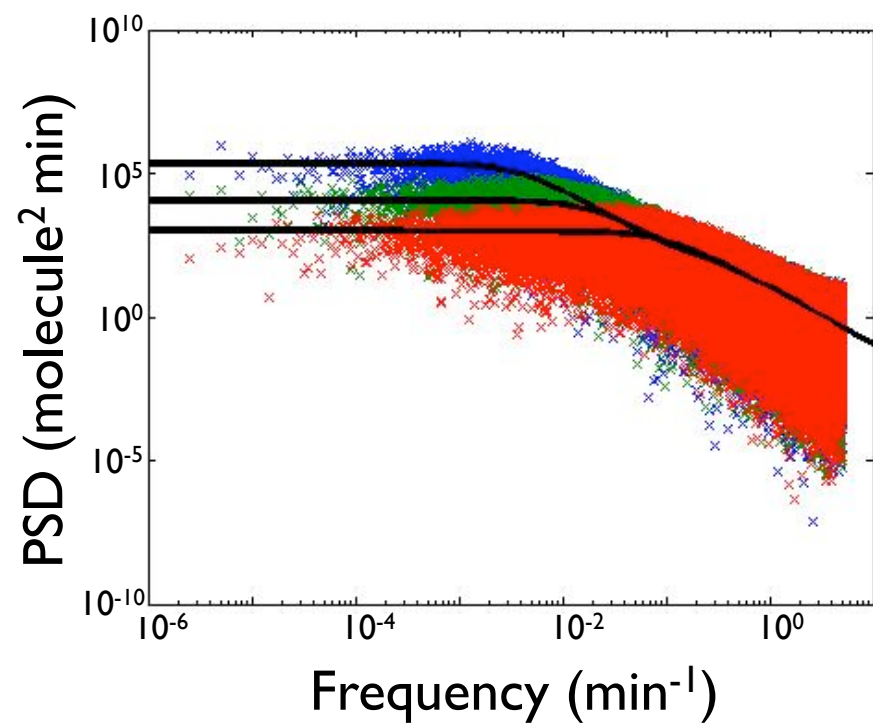

Supplement: Figure S7 — Representative results of noise and PSDs of C calculated from numerical simulations (Equation 1). Time series of C is obtained over a time span of 400,000 min with sampling frequency of 10 min−1. Numerical simulation is implemented as in Figure 2. (A) The square of the total noise in C (η T 2) is calculated from the time series (red dots). The blue line indicates η T 2 calculated by the analytical approach. For these calculations, γR = 0.2 min−1. (B) The PSDs are calculated by taking absolute values of fast Fourier transformation of the time series. For these calculations, P = 2×10^−13^ L min−1, γR = 0.02 (blue), 0.2 (green), or 2 min−1 (red). The black lines indicate PSD calculated by the analytical approach. (0.06 MB PDF) [file pcbi.1000167.s008.pdf]

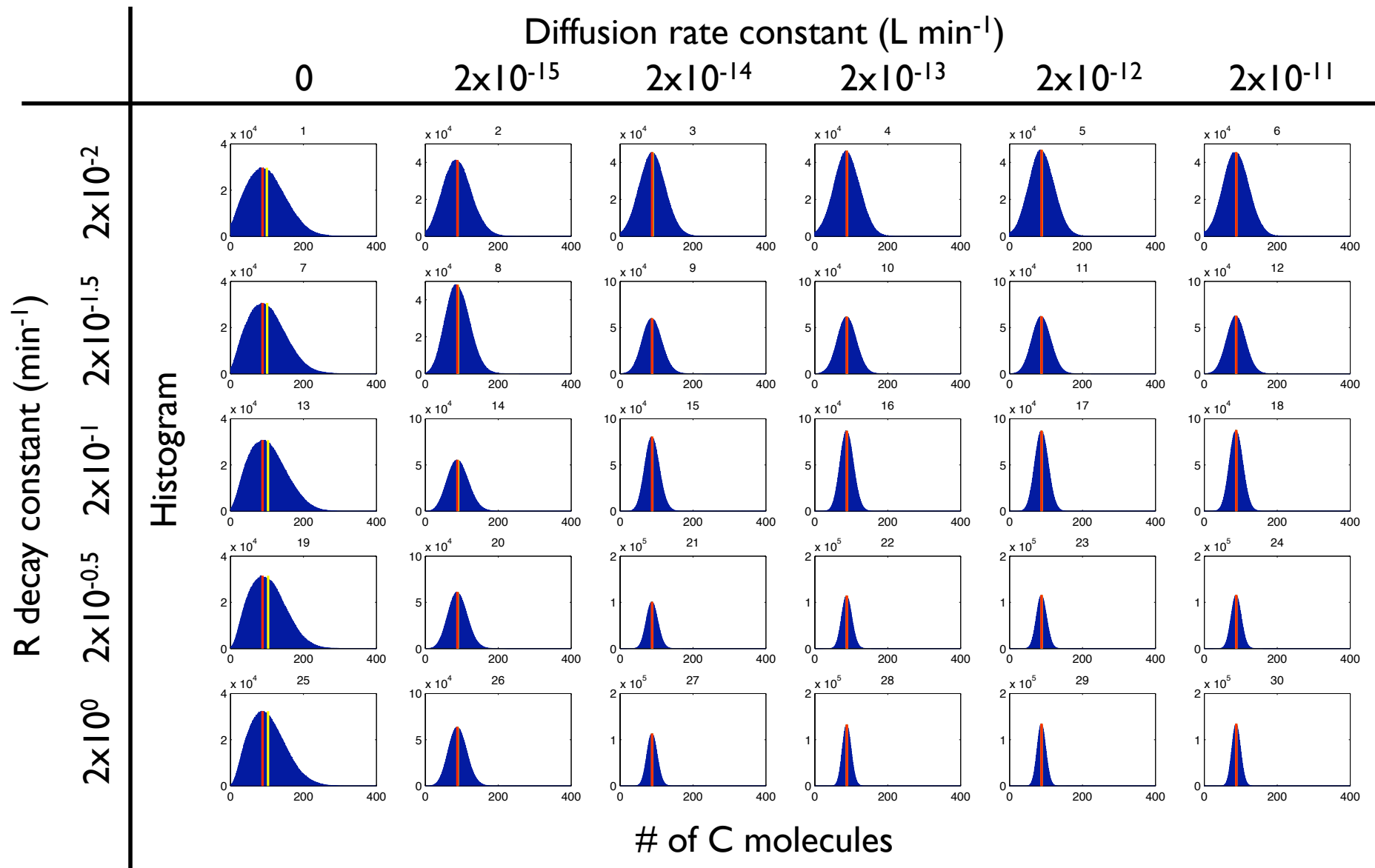

Supplement: Figure S8 — Simulated histograms of C using Equation 1 for varying P and γR. A red line indicates the steady-state value of C calculated by the deterministic version of Equation 1, whereas a yellow line indicates the mean value of the corresponding distribution. Numerical simulation is implemented as in Figure 2. (0.52 MB PDF) [file pcbi.1000167.s009.pdf]
