# Supplementary material for: Noise Reduction by Diffusional Dissipation in a Minimal Quorum Sensing Motif
Source: PLoS Comput Biol. 2008 Aug 29;4(8):e1000167. doi: 10.1371/journal.pcbi.1000167 (PMC2507755; doi:10.1371/journal.pcbi.1000167)
Supplement: Table S2 — Base parameter values. (0.05 MB PDF) [file pcbi.1000167.s011.pdf]

Table S2: Base parameter values

Elementary Reaction	Parameter	Definition	Value	Reference
$* \rightarrow A_i$	k_A	Internal AHL production rate constant	842 molecules min^{-1}	Varied depending on diffusion rate to keep the steady state level constant (10 nM). 10 nM is a concentration that elicits half-maximal activation of LuxR/I system [1].
$* \rightarrow R$	k_R	R protein production rate constant	20 molecules min^{-1}	Set to provide a saturating level of R protein.
$R + A_i \rightarrow C$	k_{C1}	Complex association rate constant	0.1 $molecules^{-1}$ min^{-1}	Estimated to favor complex association because R proteins tend to form a stable complex [2].
$C \rightarrow R + A_i$	k_{C2}	Complex dissociation rate constant	1 min^{-1}	Estimated to favor complex association because R proteins tend to form a stable complex [2].
$A_i \rightarrow *$	γ_{A_i}	Internal AHL decay rate constant	0.023 min^{-1}	Dominated by dilution due to cell growth.
$A_e \rightarrow *$	γ_{A_e}	External AHL decay rate constant	0.0018 min^{-1}	Measured hydrolysis rate of 3-Oxo-C_6-AHL is 3.07×10^{-5} s^{-1} [3].
$R \rightarrow *$	γ_R	R protein decay rate constant	0.2 min^{-1}	Measured TraR half life is 3.5 min [2].
$C \rightarrow *$	γ_C	Complex decay rate constant	0.02 min^{-1}	Stable and dominated by dilution due to cell growth.
$A_i \leftrightarrow A_e$	P	AHL diffusion rate constant	2×10^{-12} L min^{-1}	Estimated from diffusion rates of sugar group [4].
	V_i	Cell volume	1.6×10^{-15} L	Typical cell volume of *E. coli* [5].
	V_e	Microenvironment volume (average extracellular volume per cell)	7.99×10^{-9} L	
	β	Effective magnitude of extrinsic noise source	49	Set so that extrinsic noise dominates total noise.
$C + C \rightarrow D$	k_{D1}	Dimer association rate constant	0.1 $molecules^{-1}$ min^{-1}	
$D \rightarrow C + C$	k_{D2}	Dimer dissociation rate constant	1 min^{-1}	

References

1. Collins CH, Arnold FH, Leadbetter JR (2005) Directed evolution of Vibrio fischeri LuxR for increased sensitivity to a broad spectrum of acyl-homoserine lactones. Molecular Microbiology 55: 712--723.
2. Zhu J, Winans SC (2001) The quorum-sensing transcriptional regulator TraR requires its cognate signaling ligand for protein folding, protease resistance, and dimerization. Proc Natl Acad Sci U S A 98: 1507--1512.
3. Kaufmann GF, Sartorio R, Lee S-H, Rogers CJ, Meijler MM, et al. (2005) Revisiting quorum sensing: Discovery of additional chemical and biological functions for 3-oxo-N-acylhomoserine lactones. Proc Natl Acad Sci U S A 102: 309--314.
4. Nikaido H, Rosenberg EY (1981) Effect on solute size on diffusion rates through the transmembrane pores of the outer membrane of Escherichia coli. J Gen Physiol 77: 121-135.
5. Prescott LM, Harley JP, Klein DA (1996) Microbiology. Dubuque, IA: Wm. C. Brown Publishers.
